# Taguchi optimization of Wire EDM process parameters for machining LM5 aluminium alloy

**DOI:** 10.1371/journal.pone.0308203

**Published:** 2024-10-28

**Authors:** Sunder Jebarose Juliyana, Jayavelu Udaya Prakash, Robert Čep, Charles Sarla Rubi, Sachin Salunkhe, Arasumugam Divya Sadhana, Emad Abouel Nasr

**Affiliations:** 1 Department of Mechanical Engineering, Vel Tech Rangarajan Dr. Sagunthala R&D Institute of Science and Technology, Avadi, Chennai, India; 2 Department of Machining, Assembly and Engineering Metrology, Faculty of Mechanical Engineering, VSB-Technical University of Ostrava, Listopadu Ostrava, Czech Republic; 3 Department of Physics, Vel Tech Rangarajan Dr. Sagunthala R&D Institute of Science and Technology, Avadi, Chennai, India; 4 Department of Biosciences, Saveetha School of Engineering, Saveetha Institute of Medical and Technical Sciences, Chennai, India; 5 Department of Mechanical Engineering, Gazi University Faculty of Engineering, Maltepe, Ankara, Turkey; 6 Department of Industrial Engineering, College of Engineering, King Saud University, Riyadh, Saudi Arabia; Ramaiah Institute of Technology, INDIA

## Abstract

LM5 alloy is suitable for metal castings for marine and aesthetic uses due to its admirable resistance to corrosion. In order to make intricate shapes in the LM5 alloy, this study intends to assess the impact of Wire Electric Discharge Machining process variables, like Pulse on Time (T_on_), Pulse off Time (T_off_), Gap Voltage (GV) and Wire Feed (WF) on responses like Material Removal Rate (MRR), Surface Roughness (SR), and Kerf Width (K_w_). The LM5 aluminium alloy plate was produced through stir casting process. SEM, EDAX and XRD images confirm the LM5 Al alloy’s microstructure and crystal structure. WEDM studies were conducted using design of experiments approach based on L_9_ orthogonal array and analysed using Taguchi’s Signal to Noise Ratio (S/N) analysis. Pulse on Time has the greatest statistical effects on MRR (68.25%), SR (79.46%) and kerf (81.97%). In order to assess the surface integrity of the WEDM machined surfaces, the SEM study on the topography was conducted using the optimum surface roughness process variables: T_on_ 110 μs, T_off_ 50 μs, GV 40 V, and WF 9 m/min. SEM images show the recast layer and its thickness. The average absolute error for MRR is 1.69%, SR is 3.89% and kerf is 0.88%, based on mathematical (linear regression) models. The Taguchi’s Signal to Noise ratio analysis is the most appropriate for single objective optimization of responses.

## Introduction

Wrought alloys and cast alloys are the two main divisions of aluminium alloys. In the first case, alloys undergo treatment in a solid state but in the later; they are liquefied in a furnace and then poured into moulds. Aluminium alloys may be categorized as heat-treatable or non-heat-treatable depending on the strengthening mechanisms used. Due to their beneficial qualities, such as their high strength-to-weight ratio, affluence of fabrication, high degree of workability, noteworthy ductility, owing thermal conductivity, strong resistance to corrosion, and appealing physical appearance at their inherent finish, aluminium alloys have become popular as structural materials over the past few years [1]. Because of this, the marine industry currently uses 25% of the world’s aluminium manufacturing.

The thin, malleable metal aluminium has great plasticity, acceptable weldability, sufficient tensile and compressive strength, and extremely significant thermal and electrical conductivity. The electronics industries use it frequently. In the automotive, marine, and aerospace industries, where weight and mechanical qualities are prioritized, aluminium alloys are primarily employed. Aluminium alloy’s machinability is far worse than pure aluminium. The degree of strain hardening, soft particles, and precipitates has each a positive impact on an alloy’s capacity to be machined [2]. Nevertheless, aluminium alloys are regarded as tough to machine materials, especially for dry machining, not withstanding their mechanical qualities. Its high heat conductivity, low melting point and propensity to stick to the cutting edges of tool materials are difficulties. In addition to the high thermal conductivity of aluminium, which removes a significant amount of heat from the cutting edge into the work piece throughout the machining process, the material is thermally deformed. Because of the low melting point of aluminium alloys, there are issues with chip development, chip elimination, and material clinging to the cutting tool [3]. So it would appear to be quite beneficial to utilize an innovative way while cutting aluminium. Due to aluminium’s strong electrical conductivity, WEDM technique should be appropriate [4].

In order to remove material, WEDM technology uses thermoelectric energy amongst the work piece and a wire electrode. The pulse discharging takes away the material from the work piece by melting and evaporating it in a tiny space separating the work piece and the electrode. This technique is typically employed to produce intricate shapes and to manufacture materials that are challenging for regular equipment to work with [5]. EDM is superior to traditional machining in many ways. Electrically conductive materials can be cut using EDM, and this technique has been used to machine work pieces that have been heat-treated and hardened. Intricate and complex profiles can be cut more quickly, precisely, and affordably. Burrs have been avoided and thin, delicate parts have been created with ease. The mechanical properties of the material have no bearing on the WEDM cutting process; the only need is to achieve the bare minimum conductivity of the processed material. Because of its great accuracy and minimum surface roughness, this technology is suited for cutting very hard conductive materials, composites, ceramics, or sandwiches. Considering the significance of WEDM manufacturing, it must be less expensive than traditional machining in order to be successful. In general, the use of electrical discharges represents a trade-off between productivity and machining quality. Numerous factors might have an impact on the WEDM cutting process [6]. Each one of them has a different impact on the price of production and the work piece’s final grade finish. Thus, applying DoE should be beneficial. Schedule of test and statistical analysis of specified plan make up the DoE (Design of Experiments), very effective. The conclusion of a planned experiment’s evaluation is whether the variables under observation were affected by the tested elements. An experiment’s output is a particular value of the observable variable, also known as the dependent variable or response, which describes the test’s quality. The ultimate quality is affected by a wide range of factors. Under the perspective of experimental design, they can be classified into specific and randomized. WEDM machine parameters are the inputs to the procedure of machining. Just those factors which have a statistically noteworthy outcome on the degree of quality should be ultimately chosen after thoroughly examining all components and their common interactions. It enables the variables that matter most to be set at their ideal values while also identifying the unnecessary variables, lowering their tolerance and thereby lowering manufacturing costs [7, 8].

Research on WEDM on Al6061 alloy show that EDM, a nonconventional machining method, is superior in terms of dimensional accuracy and precision; when it comes to cutting hard, conductive materials. In EDM, sparks repeatedly discharge when voltage is applied after both electrodes have been dipped in dielectric. These sparks flush away the material from the surface as melted and degraded particles that are removed by dielectric. Researchers have taken into consideration advancements and modifications to the EDM process over time. A recent invention to improve the EDM process’s competency involves agglomerating powder with both an external magnetic field and a dielectric. This procedure demonstrates the ability to machine complex and sophisticated 3D profiles with reduced tool wear rate (TWR), increased productivity, and better surface quality and precision. The MFAPM-EDM technique is used in the industrial, aerospace, automotive, defense, and surgical industries for the machining of different components. In the PMEDM process, the powder particles’ accumulation of charges causes several sparks to occur during machining. More charges are produced when ions from powder particles collide with dielectric molecules due to the accumulated charge in the machining area. Multiple sparks cause the surface materials to be removed more quickly and create shallow craters, which improved MRR and SR [9].

The feasibility of utilizing the Maglev EDM for machining aluminium 6062 alloy is assessed and examined. The Maglev EDM achieves tool location by means of a methodical combination of magnetic repulsive forces. Brass tools were used in the experiments, and the surrounding air served as a dielectric for the Al-6062 alloy. The data from the previously published literature was further contrasted with the experimental results. Field-emitting SEM investigation of the machined surface revealed the presence of recast layers, globules, lumps of debris, melted debris, micro-pores, micro-voids, micro-cracks, and craters [10].

WEDM’s essential indicators of performance are MRR, SR, and kerf. The MRR in WEDM processes determines the cost of machining and the rate of output. The primary purpose when establishing the machining parameters is to maximize MRR and minimize SR. The Taguchi technique, a powerful experimental design tool, takes a simple, effective, and systematic approach to determining the ideal machining parameters. Furthermore, this strategy has a low experimental cost and effectively reduces the effect of the source of variation. An economical and simple technology for modifying machined surfaces while maintaining accuracy must be developed [11].

The main objective of this study is to optimize the WEDM process parameters for machining LM5 aluminium alloy using Taguchi’s Signal to Noise ratio (S/N) analysis. Other objective is to study the effect of machining parameters on the MRR, SR and kerf. Another objective is to construct Mathematical models using regression equations and to find the deviation % between the experimental and predicted values of MRR, SR and kerf. The machined surfaces were analyzed under SEM to find the recast layer.

## Literature survey

Numerous researchers adjusted the settings of several machines to produce high-quality products [12]. Various techniques have been used in past to measure how control parameters affect part surface characteristics, MRR and K_w_. The unstable wire portion is the main cause of difficulties [13]. To ascertain the relationship between machining performance and machining parameters, an experimental analysis of the wire breakage phenomenon using a thermal model was conducted. With a reduction in T_off_, the MRR initially rises [14]. However, the gap becomes unstable after a relatively brief period of time, which lowers the MRR. As the MRR rises, surface quality declines. K_w_ and SR have been found to be significantly influenced by the T_on_ and the D_ww_. Additionally, it has been discovered that using a medium D_ww_ can improve SR; modifying T_on_ and D_ww_ can regulate final cutting.

It was discovered that by reducing I, T_on_ and T_off_, surface roughness can be improved. Shorter and long pulses will create a comparable SR but differing surface morphologies and MRR. In comparison to long pulse duration, the clearance rate is significantly higher under short pulse duration. Furthermore, compared to a long pulse, a short pulse can produce better SR [15].

Numerous criteria and difficult experimental effort are needed for the study of the WEDM process, which takes a greater amount of money and time. Therefore, it is vital to optimize the control parameters to decrease the money and time required to assess the WEDM process. There is research on experiment design that enables a method to show the effect of control factors with the least amount of experiments. Some investigations use fractional factorial designs, while others use full-factorial designs to evaluate variables with impact [16].

Coated wires are chosen so as to achieve homogeneous surface qualities. Most delicate characteristic that affects the creation of a layer made up of a combination of oxides is the T_on_ and T_off_. The development of oxides can be significantly reduced by reducing the duration between two pulses [17].

Research of Kansal et al [18] shows in PMEDM, the electrically conductive powder is mixed in the dielectric of EDM, which reduces the insulating strength of the dielectric fluid and increases the spark gap between the tool and work piece. As a result, the process becomes more stable, thereby, improving the material removal rate (MRR) and surface finish. The powder particles simplify the igniting process by increasing the spark gap and reducing the insulating strength of the dielectric fluid.

The WEDM process’s performance for titanium alloys was the focus of investigation of Debnath and Patowar [19]. Based on Taguchi DoEs, the results show that the flushing pressure of dielectric, wire tension, and T_on_ are important process parameters that have a considerable impact on the machined hole’s circularity, cylindricity, and diametral errors, since wire tension affects both stability and wire electrode rigidity. This study does not include the MRR and SR, which are the most vital process responses.

In the case of MMCs, T_on_ is the most important component for K_w_ [20]. Taguchi’s DoE based orthogonal arrays should be used instead of full factorial experiments.

Sahoo et al. [21] used EDM technology to analyze titanium diamond machining performance measures. I_p_ and T_on_ are the chosen control variables, and during experimental runs, a duty factor of 50% to 75% is maintained. R_a_, TWR and MRR are the responses taken into account for this operation. The analysis has been carried out using L_16_ OA. In order to find the ideal input parametric combination, analysis of variance is also used in conjunction with overall evaluation criteria (OEC). The findings of the analysis indicate that T_on_ has a greater influence on TWR than does current on MRR and R_a_. Additionally, the identification of various material phases on a machined work surface is aided by EDX, SEM and XRD.

For tool positioning, Maglev EDM uses a novel bipolar linear self-servo technique that produces consistent machining stability using a special magnetic repulsive force balance action. The performance characteristics of each dielectric have been investigated. The evaluation of surface morphology shows that the use of bio-dielectrics has the potential to significantly improve surface uniformity and reduce deformities [22, 23].

For the processing of nanosecond pulsed lasers, a unique and straightforward model based on geometric mathematics and heat transport was proposed. Experiments verify that the models are feasible. The single-pulse laser ablation craters had errors in both diameter and depth of 2.56% - 7.14% and 6.82% - 18.91%, respectively. Between 3.47% and 12.47% is the recast layer depth error for consecutive stacked ablation pulses. On metal surfaces ablated using pulsed laser, it forecasts the shape of the recast layer. The model serves as a guide for the preparation of surface functionality [24].

Based on the extensive literature survey, the authors of this article were persuaded to carry out a research study to recommend the most optimal machining parameters for efficiently machining stir-casted LM5 Aluminium alloy in order to achieve maximum MRR, minimum SR and minimum Kerf using Pulse on Time (T_on_), Pulse off Time (T_off_), Gap Voltage (GV) and Wire Feed (WF) at three levels, which have not been reported before by any researcher.

This study also provides a general overview of a thorough process to determine the best machining parameter settings based on the design of experiments approach. The research paper indeed aims the mathematical models that are constructed to associate the machining response characteristics with machining control parameters, in addition to revealing the results of signal/noise (S/N) ratio analysis and ANOVA. To better understand the occurrence of recast layer generation during machining, energy dispersive spectroscopy (EDS) analysis and scanning electron microscopy (SEM) analysis were used to examine the machined surface textures.

## Materials and methods

### LM5 aluminium alloy

The LM5 aluminium alloy has excellent casting properties, sturdy structure, and great durability against corrosion. The material is commonly utilized in the automotive, aerospace, and marine sectors, whenever a combination of properties is required [25, 26]. Since it is easy to grind, weld, and cast into complex shapes, Machinery require extremely strong resistance to corrosion from sea water or marine atmospheres, along with castings which need to display and maintain a high polish Gestalt, all have uses for LM5 aluminium [27, 28]. The chemical composition of LM5 Aluminium Alloy using Optical Emission Spectrometry (ASTM E 1251–07) is shown in Table 1.

**Table 1:** Chemical composition of LM5 aluminium alloy.

Cu	Mg	Si	Mn	Fe	Pb	Zn	Al
0.032	3.299	0.212	0.022	0.268	0.02	0.01	Balance

### Fabrication

**Fig 1:**
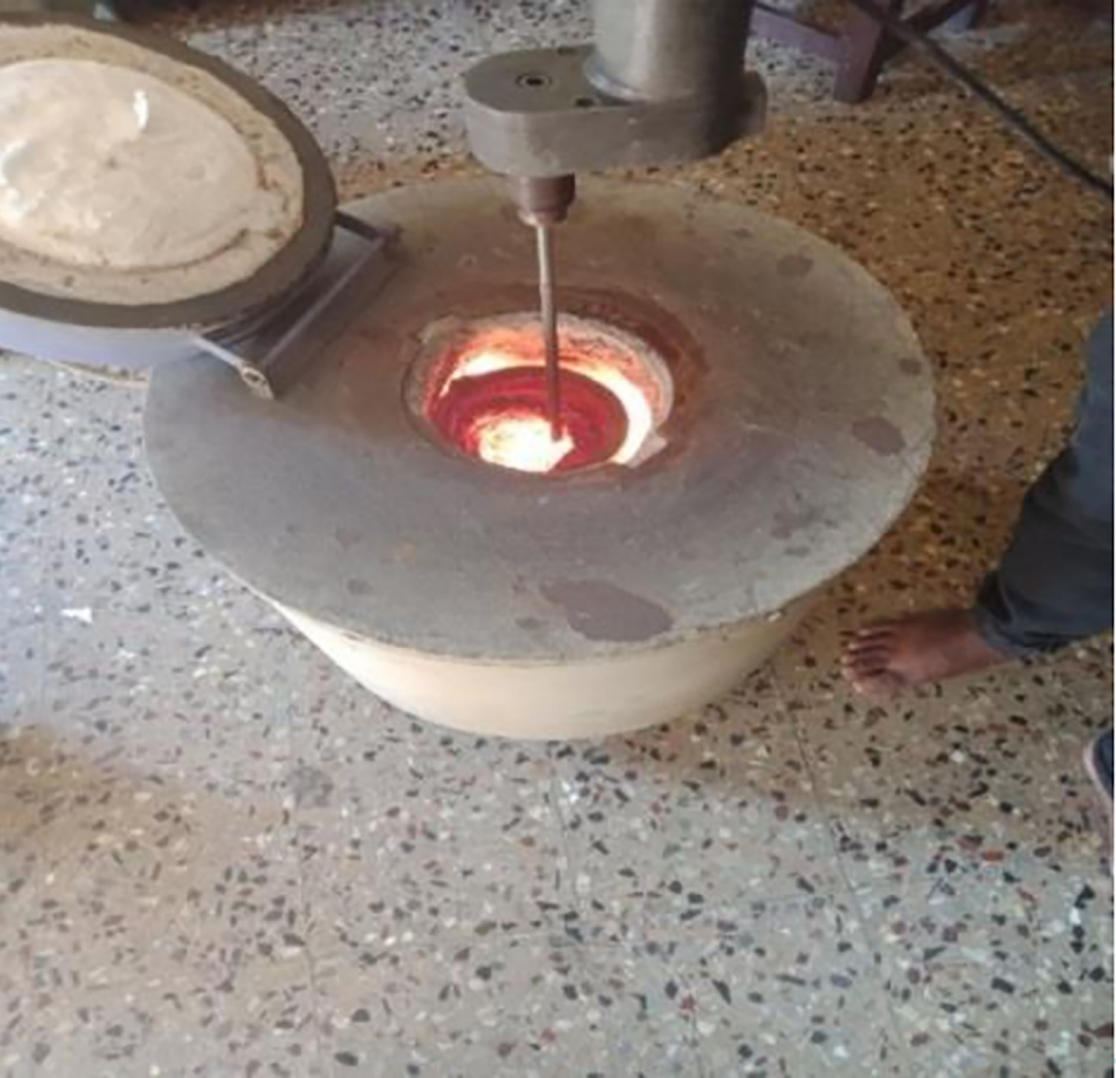
Stir casting setup.

Stir casting process was used to manufacture plates of the LM5 Al alloy, measuring 120*120*10 mm. Easy use, inexpensive manufacturing, a uniform dispersion of reinforcing elements, and improved mechanical qualities are only a few benefits of stir casting. Fig 1 depicts the stircasting setup.

A graphite-coated container served to melt LM5 alloy ingots in a furnace powered by electricity. To 850°C, the ambient temperature was raised steadily. The liquid state of the melt at 800°C was degassed using hexachloroethane. The molten metal was subsequently fed into preheated (650°C) cast-iron moulds after being agitated at 600 rpm for 10 minutes [29, 30].

### Micro-structural analysis

**Fig 2:**
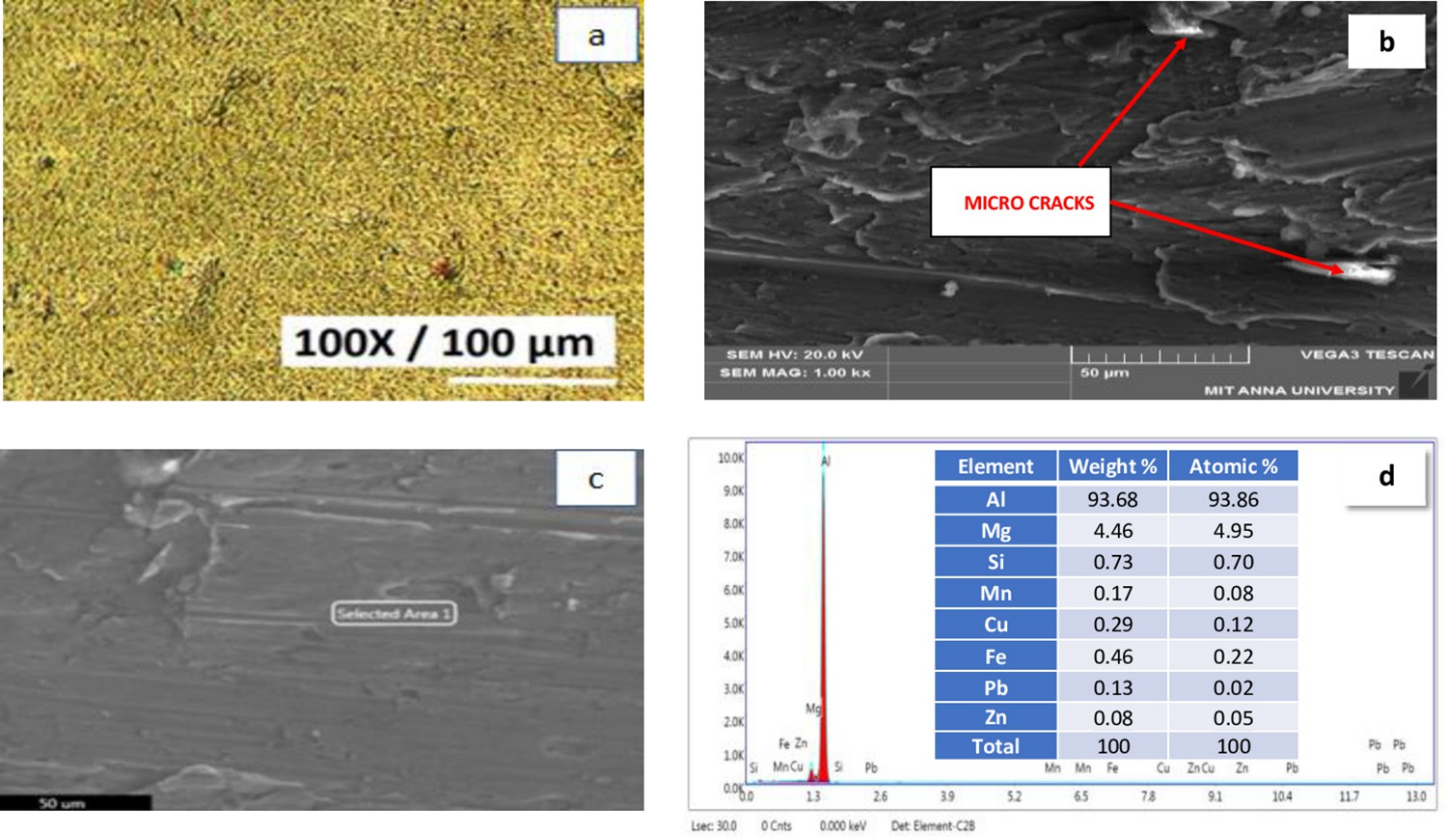
LM5 Aluminium alloy a) Optical Micro graphs b) SEM c) SEM of selected area d) EDAX.

Fig 2A shows the Optical Micro graph of LM5 aluminium alloy Fig 2B shows the SEM image of LM5 Fig 2C and 2D shows the SEM of selected area and EDAX respectively.

Primary aluminum grain interdendritic pattern is visible in the microstructure. MgAl_2_ eutectic particles that were not dissolved during solidification precipitate near the grain boundaries [31]. The grain size of the primary aluminium phase is 40–50 microns. The magnification is 100x. Scanning electron microscopy (SEM) was utilized to collect data from a mix of backscattered electron (BSE) and secondary electron (SE) signals. The BSE signal emphasizes compositional difference, whereas the SE signal depicts sample topography, including cracks and voids. Thermomechanical processing and alloying influence the development of iron-rich intermetallic particles and can be tuned to reduce the likelihood of micro-cracking [32]. Figure depicts micro-cracks in an iron-rich intermetallic particle. These endogenous micro cracks can enlarge as the material is bent into its final shape, resulting in bigger material fractures. The scale bar is 100 μm. EDAX test results confirm that the elements like aluminium, iron, copper, magnesium, zinc, silicon, carbon, oxygen, lead and other elements are present in the aluminium alloy as shown in all the specimens, aluminium shows the peak value followed by magnesium as LM5 is magnesium based alloy. The results confirm the presence of Al (high-intensity peaks).

### Experimental analysis on machinability of LM5 Al alloy by WEDM

Aluminium alloy was used to prepare the test samples. The experimentation was done using the ECOCUT WEDM, which let users to select input settings based on the sample’s material and thickness. The manufacturer’s instructions should be used to select the tool material. Brass wire of 0.25 mm in diameter was used in this instance. The dielectric medium utilized was demineralized water. The worktable, the servo regulating system, the power supply, the dielectric supply system, and the wire are the fundamental components of the WEDM machine. Using common grips, the material samples were secured to the machine’s worktable.

**Fig 3:**
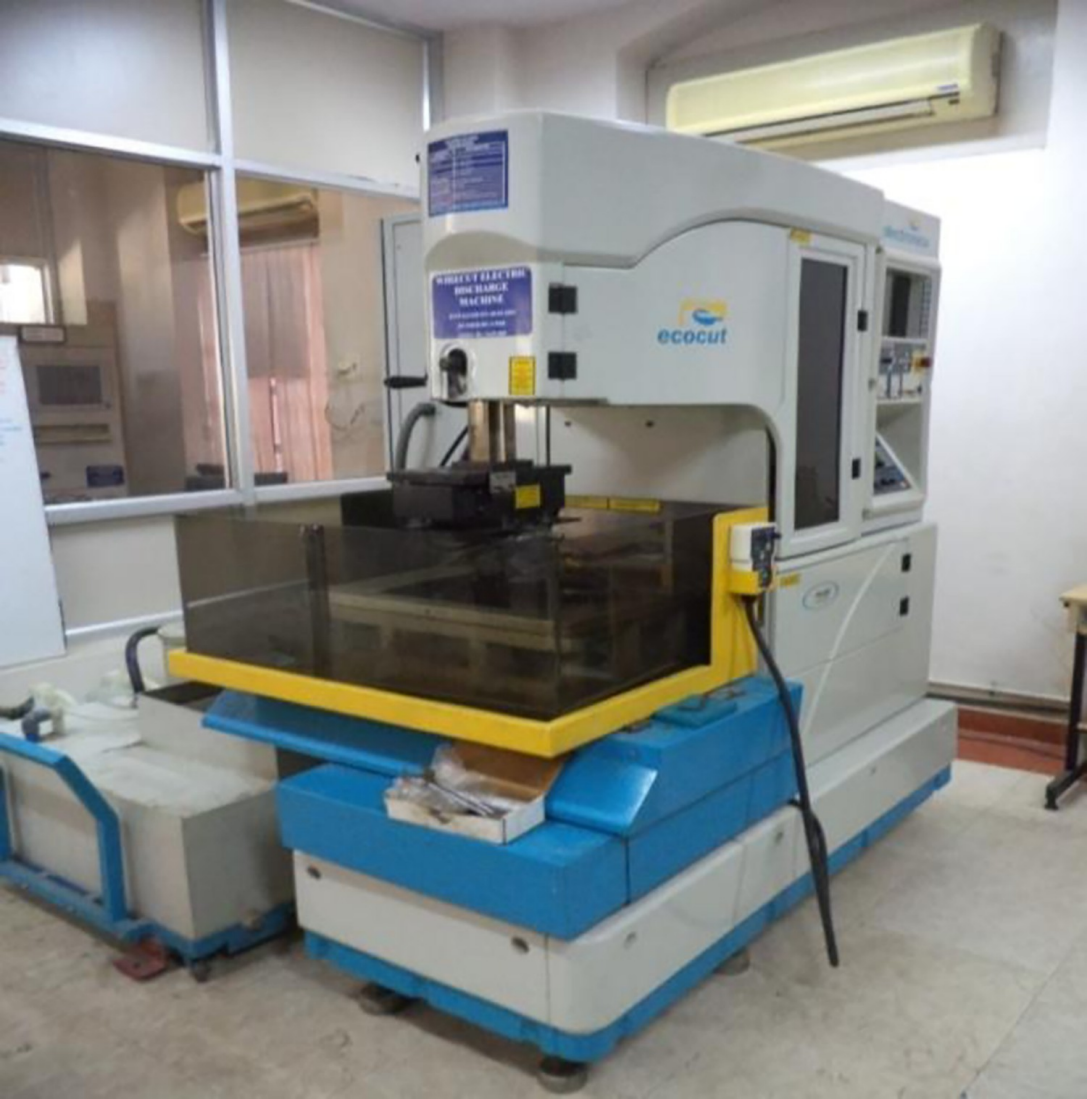
Photograph of ECOCUT–CNC wire EDM.

WEDM removes material using a sequence of recurrent spark discharges across the tool (wire electrode) and work piece, which are submerged in a liquid dielectric and isolated by a distance known as the spark gap. Whenever a suitable voltage is given during pulse-on time, the dielectric breaks down, causing an electrical spark to form between the tool and workpiece. Thermal conduction converts electrical energy into heat energy by means of the creation of a discharge column. The tool and workpiece begin to melt as a result of high-energy plasma production. While the discharge begins, the tool, work piece, and dielectric begin to vaporize, resulting in the development of a compressed vapor bubble which increases till the pulse-on time. At the start of the pulse-off period, the discharge stops, resulting in a dramatic implosion of the plasma channel and squeezed vapor bubble, allowing the superheated and molten liquid to explode into the dielectric. The ejected materials re-solidify into small spheres, which are washed away by the dielectric. This caused the creation of a small cavity or crater on the work piece surface. With each discharge, the needed amount of material is removed from the work piece surface [33]. The WEDM process is chosen for studying the machinability of the LM5 aluminium alloy. The photograph of CNC WEDM is presented in Fig 3.

### Design of Experiments (DoE)

The Design of Experiments (DoE) technique is used to specify what information, in what amount, and under what conditions needs to be gathered during an experiment in order to meet two primary objectives: a lower cost and greater statistical precision for the response parameters. Four distinct process parameters at three levels were chosen for the current investigation: wire feed (WF), gap voltage (GV), pulse on time (T_on_), and pulse off time (T_off_). Material removal rate (MRR), surface roughness (SR), and cutting width (kerf) are the responses. For this research, the L_9_ orthogonal array is chosen based on the parameters that were chosen. For every experimental condition, three repetitions of the experiment have been conducted. Table 2 shows the machining variables together with their respective levels.

**Table 2:** Process parameters and levels.

Level	Pulse on Time (μs) T_on_	Pulse off Time (μs) T_off_	Gap Voltage (V) GV	Wire Feed (m/min) WF
1	110	30	20	3
2	115	40	30	6
3	120	50	40	9

### Machining performance variables (Responses)

**Fig 4:**
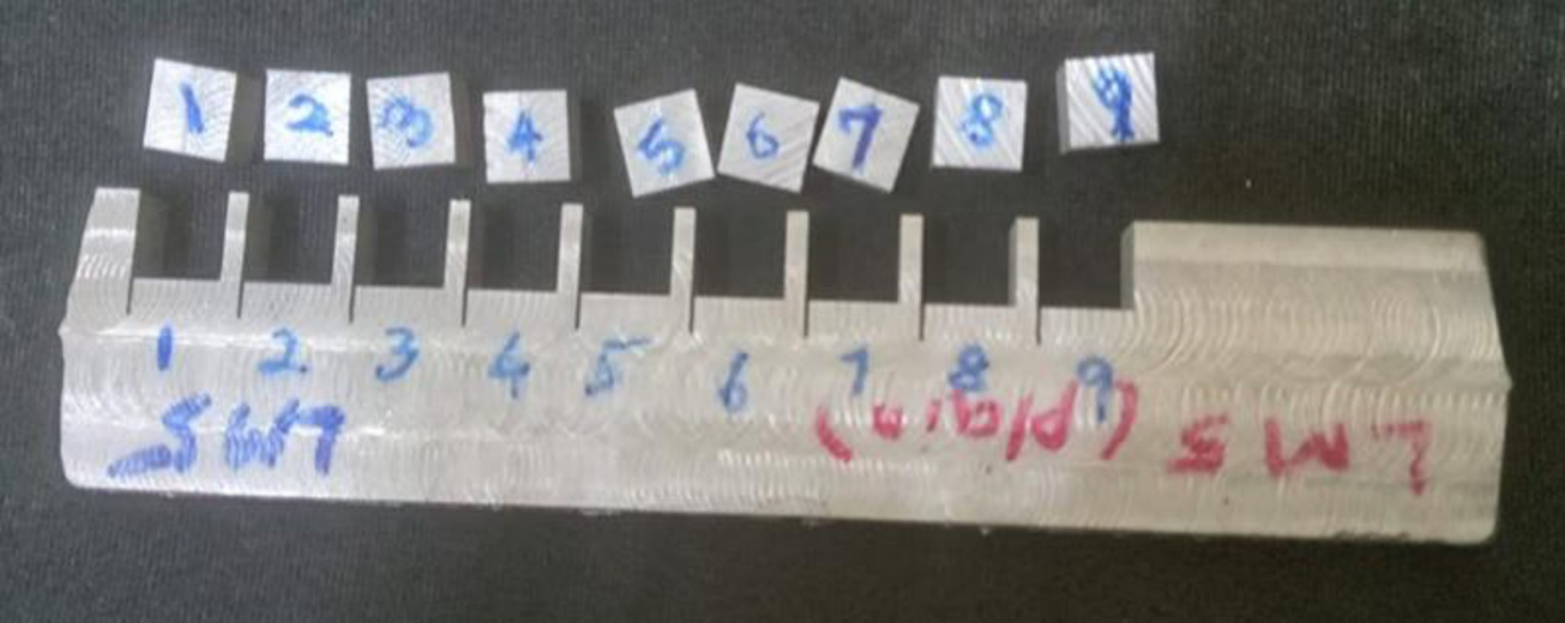
Machined specimens.

The basic objective is to establish the machining parameters to achieve the maximum MRR, the smallest possible K_w_, and the lowest possible SR. Fig 4 shows the wire Electrical Discharge machined samples.

#### Material Removal Rate (MRR)

The material removal rate (MRR) is calculated as the volume of material eliminated from the specimen (mm^3^) divided by the time taken (min).

#### Surface roughness

**Fig 5:**
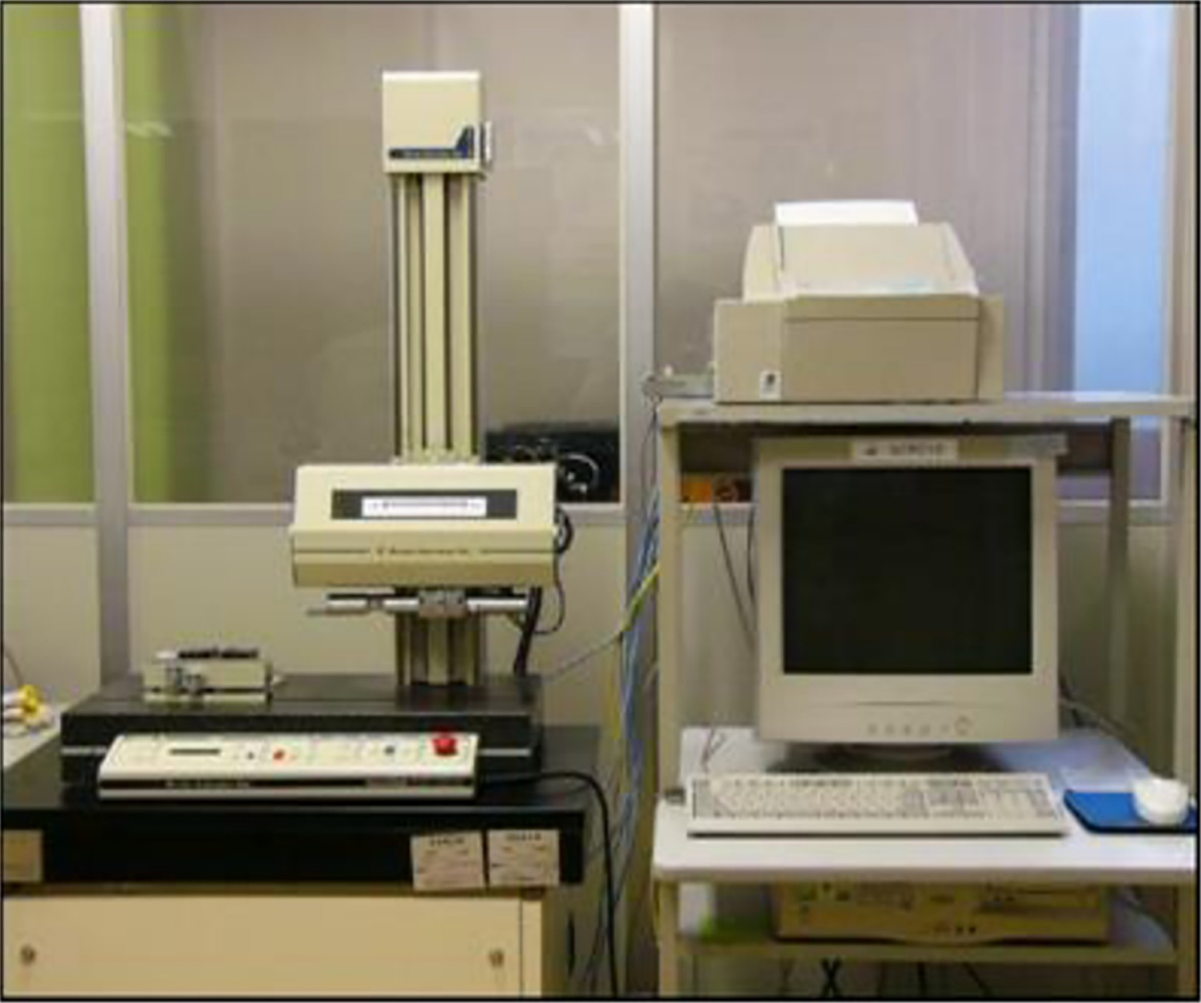
Surface roughness tester.

According to Kousik-Kumaar et al. [34] surface roughness assessment is crucial for a number of basic problems, such as friction, surface deformation, transfer of heat, current flow, stiffness of joints, and spatial precision. Three distinct sites on the surface that was machined were used to get the data, and the average of the three readings was used to determine the SR. The orientation of an SR computation was orthogonal to the surface being machined. The Surfcorder SE 3500 surface roughness tester is displayed in Fig 5.

**Fig 6:**
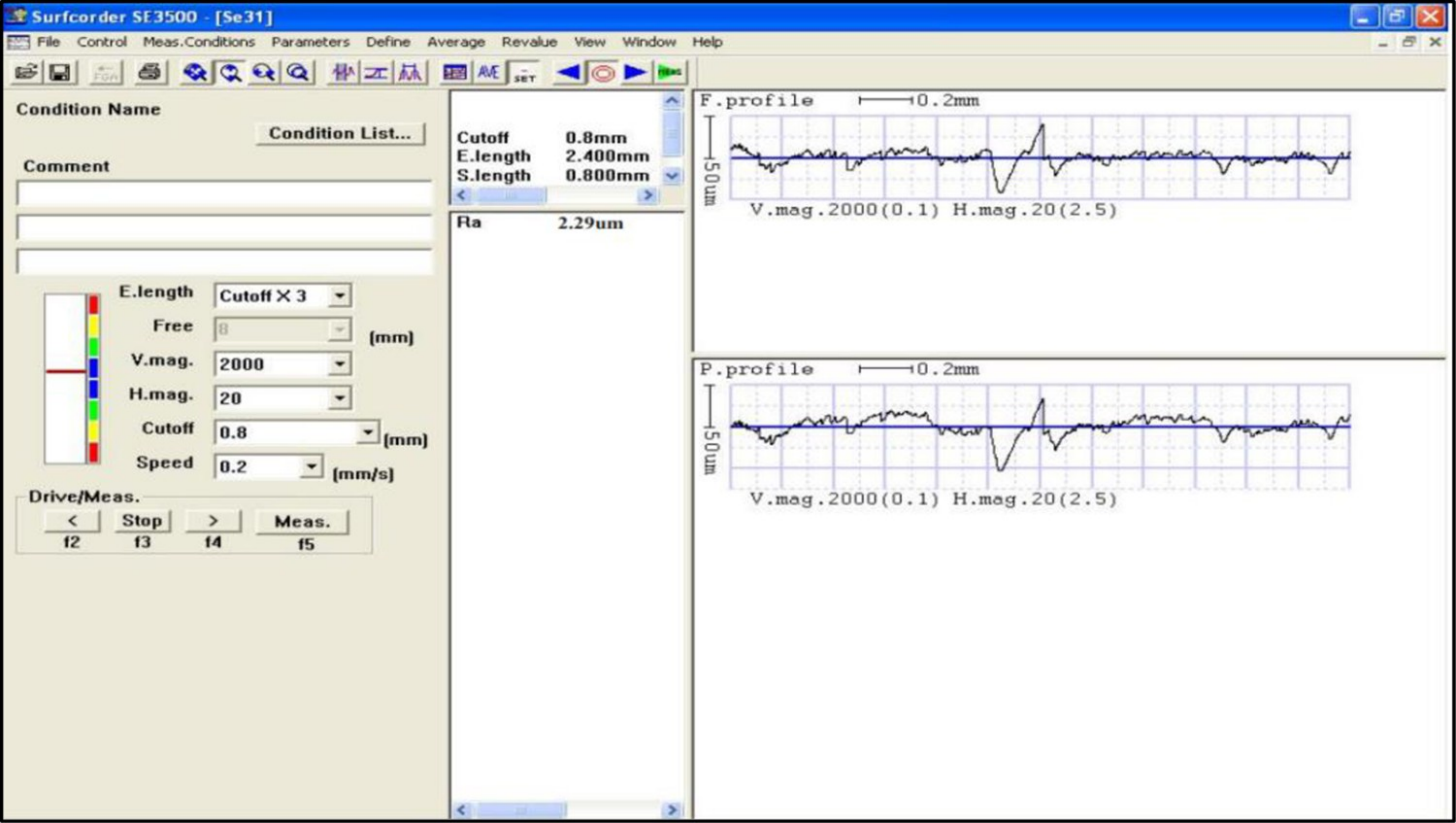
Output of surface roughness measurement.

Ra (roughness average) is a commonly used metric for quantifying a material’s surface roughness. It is the arithmetic average of the absolute values of profile height deviations from the centerline measured within a given evaluation length. The work piece was positioned vertically, with the WEDMed specimens’ axes oriented horizontally. The roughness tester was placed in front of each machined surface, allowing the stylus arm with the probe tip to be placed on the WEDMed surface. Fig 6 depicts the output shown during the measurement of surface roughness.

#### Cutting width (kerf)

The width of material which is machined by a cutting procedure is referred to as Cutting Width (Kerf). When it comes to CNC Wire EDM form cutting with traditional cutting techniques, kerf refers to the amount of material removed as the process cuts through the plate. It is measured by the help of Vision Measuring Machine. The most crucial sign of WEDM technology success is the width of the kerf in conjunction with the rate of material removal.

## Results and discussion

To ascertain the impact of machining input parameters on performance measures, experiments on wire EDM were conducted based on the Taguchi DOE approach and analyzed using S/N ratio analysis [35]. The results and pertinent factors are presented. This section converses the experimental findings related to the Wire EDM of the LM5 Aluminium alloy. The analysis and discussion focus on MRR, SR and k_W_. The ideal machining parameters were established using Taguchi’s S/N analysis and confirmation experiments to validate the results.

### Experimental results

Wire EDM experiments were conducted using Taguchi’s DoE and analyzed by S/N analysis. Table 3 displays the Wire EDM experimental results of the effect of process variables such as T_on_, T_off_, GV, WFon the responses like MRR, SR, and k_w_.

**Table 3:** WEDM experimental results of LM5 aluminium alloy.

Ex No	A	B	C	D	MRR (mm^3^/min)	S/N of MRR	Surface Roughness (μm)	S/N of SR	Kerf (mm)	S/N Kerf
	Pulse on Time (μs)	Pulse off Time (μs)	Gap Voltage (V)	Wire Feed (m/min)						
1	110	30	20	3	3.61	11.16	3.10	-9.8	0.286	10.87
2	110	40	30	6	2.98	9.50	3.27	-10.3	0.286	10.87
3	110	50	40	9	2.26	7.07	2.76	-8.8	0.286	10.87
4	115	30	30	9	4.50	13.07	3.63	-11.2	0.304	10.34
5	115	40	40	3	3.40	10.64	3.45	-10.8	0.295	10.60
6	115	50	20	6	5.52	14.84	3.66	-11.3	0.313	10.09
7	120	30	40	6	4.56	13.18	3.83	-11.7	0.304	10.34
8	120	40	20	9	7.09	17.01	3.63	-11.2	0.313	10.09
9	120	50	30	3	5.78	15.24	3.96	-12.0	0.313	10.09

### Analysis and discussion of results of MRR

**Fig 7:**
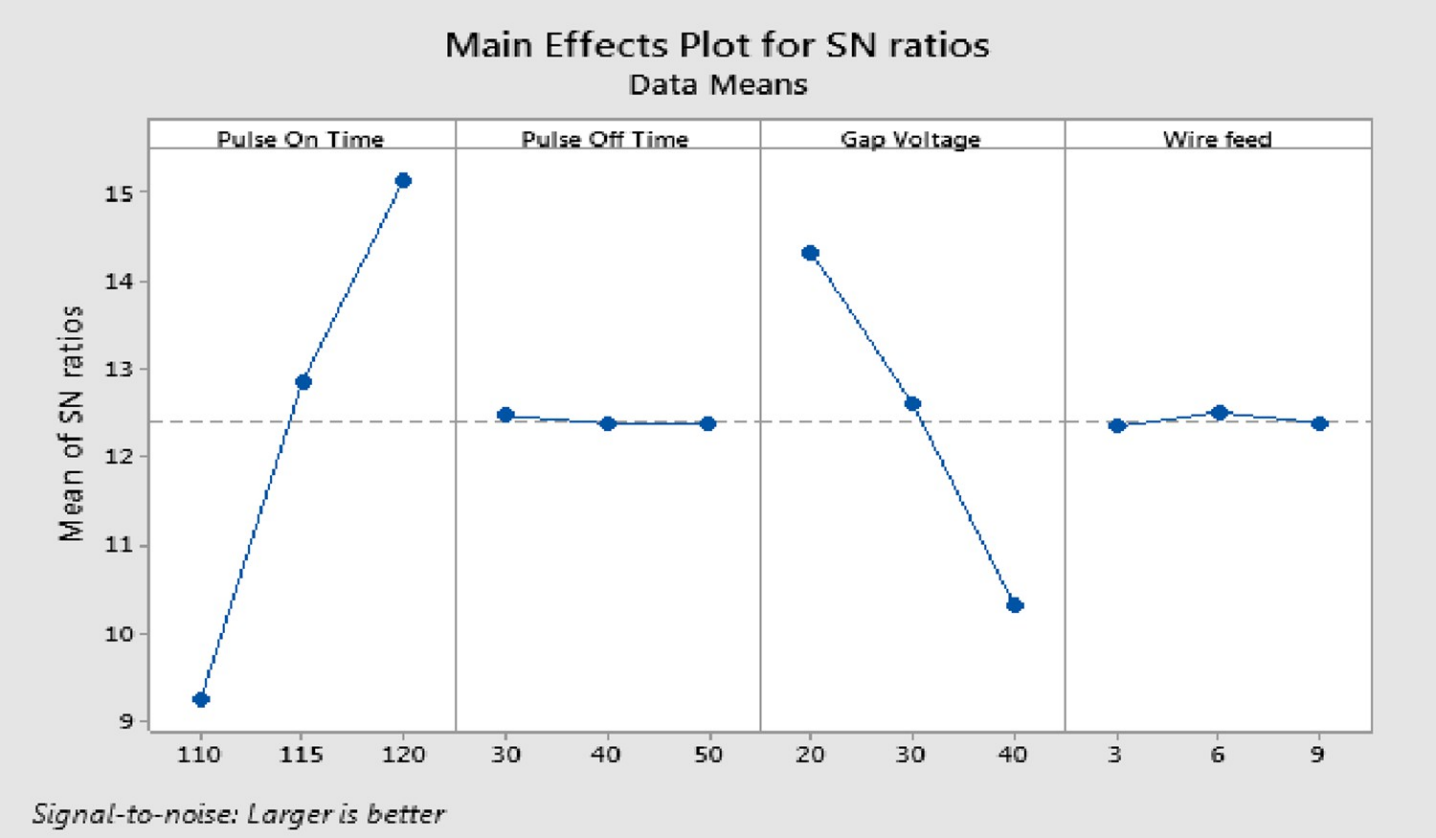
Response graphs for MRR.

S/N ratio is a metric for evaluating quality attributes. Smaller is better, nominal the best, and larger is better are three elements of performance qualities that Taguchi identified. The bigger amount of MRR is found to be more advantageous for surplus product quality, hence the "larger is better" class is chosen for S/N calculation. The ideal machining variables are estimated at the level where each variable has the highest S/N value. This section discusses the implications of the Wire EDM process settings on MRR. The S/N ratio of response characteristics for each variable at different phases is quantified using experimental results. The parametric influences on response characteristics were examined using the main effects plot (response graphs). Analysis of variance (ANOVA) was applied to S/N data in order to categorize pertinent variables and assess the effects of those variables on response characteristics. The optimum process variables are obtained by analyzing the response graphs and ANOVA table. As shown in Fig 7, the MRR rises with increasing T_on_ and decreases with rising T_off_ and GV. This occurs as a result of the discharge energy increases brought on by the increase in T_on_, which leads to a greater MRR. As the T_off_ decreases, more discharges occur in a given period of time, increasing the MRR. Lower MRR is caused by the normal discharge gap widening as the gap voltage increases [36].

### Selection of optimal levels for MRR

The delta values and ranks indicate that T_on_ has the greatest effect on MRR, followed by GV. Fig 7 and Table 4 demonstrates that the third level of T_on_, first level of T_off_, first level of GV and second level of WF produce the maximum MRR. R^2^ value is 99.93%, which is most desirable. The p-value for T_on_ and GV is below 0.05, demonstrating the significance of the factors, but the p-value for T_off_ and WF is greater than 0.05, demonstrating the meager impact of these parameters on MRR.

**Table 4:** Response table for MRR.

Level	Pulse on Time	Pulse off Time	Gap Voltage	Wire Feed
1	9.24	**12.47**	**14.34**	12.34
2	12.85	12.38	12.60	**12.51**
3	**15.14**	12.39	10.30	12.39
Delta	5.90	0.09	4.04	0.16
Rank	1	4	2	3

According to the F-test, the effect is determined to be considerable if the computed value of the F-ratio is higher than the tabulated F-value. The table’s F-value at the 5% level of significance is F_(0.05, 2,4)_ = 6.944. So, T_on_ and GV are important process parameters for obtaining larger MRR as seen in ANOVA Table 5. T_off_ and WF are two variables that have very minimal contribution on the MRR, so they are pooled up with error.

**Table 5:** ANOVA for MRR.

Source of Variation	DF	SS	MS	F	P	C %
Pulse on Time	2	53.07	26.53	1836.77	0	68.25
Gap Voltage	2	24.63	12.32	852.57	0	31.68
Pooled Error	4	0.06	0.01			0.07
Total	8	77.76				100

### Analysis and discussion of results of SR

**Fig 8:**
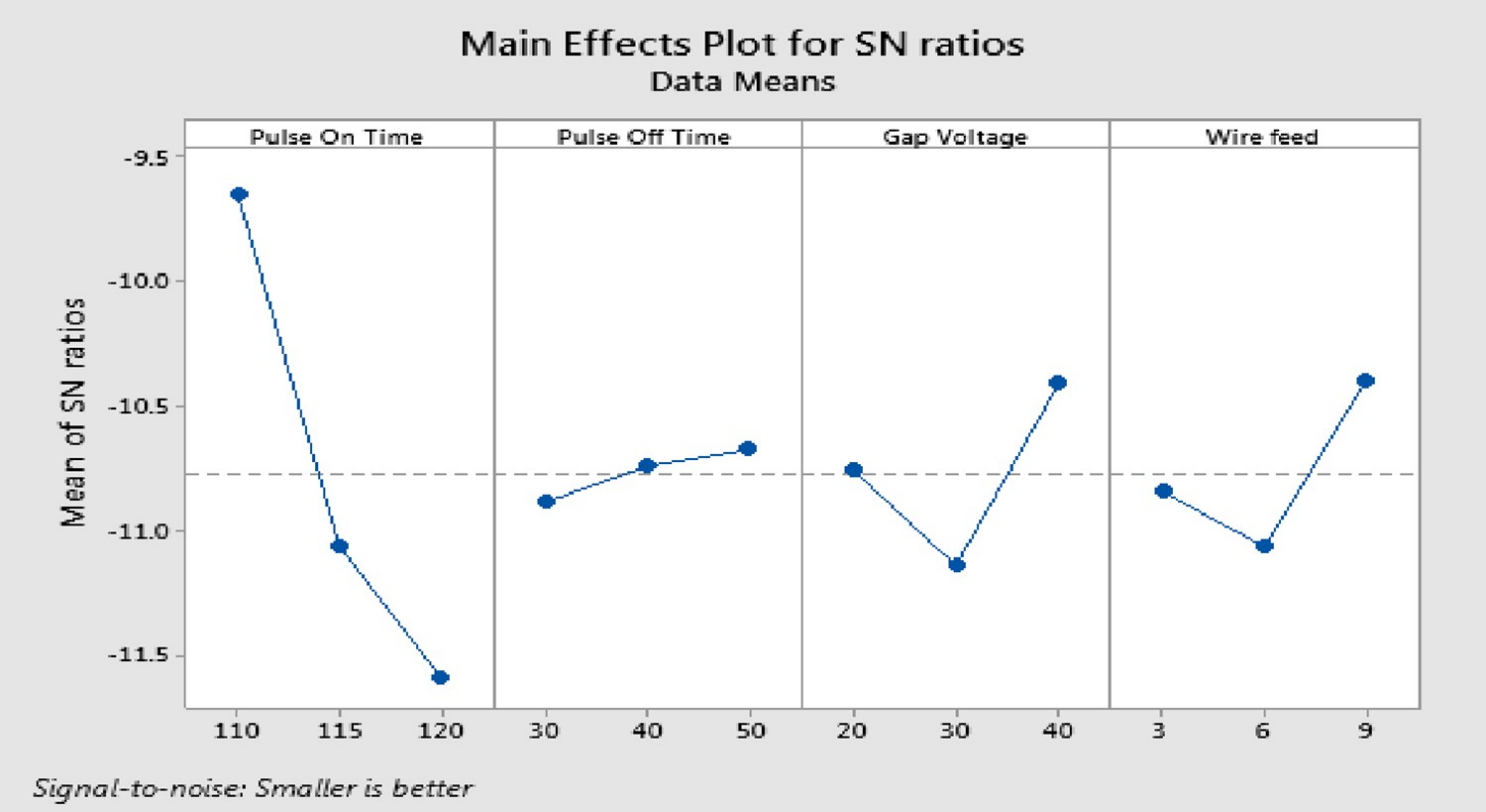
Response graphs for SR.

The "smaller is better" class is used for S/N analysis since it is found that a lesser amount of SR is better for excellent product quality. As shown in Fig 8, the SR increases with the increase in T_on_ and decreases with the increase in GV, T_off_ and WF. The cause is the discharge energy changes with T_on_ and that a higher T_on_ produces a larger crater, which raises the SR on the work piece. As T_off_ rises, the number of discharges decreases, leading to greater surface precision from steady machining. As the GV increases, the average discharge gap widens, improving SR. T_off_ has no discernible impact [37]. Following wire EDM, the surface exhibits an irregular mix of overlapping craters, micro-globules, and melted debris. During the WEMD process, the generated heat ranges between 8000 and 12,000°C which produce local melting and evaporation of the work piece material. The heat produces a high level pressure, but it is insufficient for removing all of the molten material. The balance of the molten material re-solidifies on the surface of the machined sample, resulting in an undulating topography. A larger amount of molten material re-solidifies on the machined surface, resulting in a thicker recast layer and a higher Ra.

### Selection of optimal levels for SR

For the researcher to select the ideal parameters, the experiment results are assessed. Since SR is an output characteristic where "lower is better" applies, Fig 8 and Table 6 demonstrates that the lowest SR values are found in the T_on_ first level, third level of T_off_, third level of WF, and third level of GV. The process factors significance was examined using an ANOVA. R^2^ for SR is 99.09%, which is a desirable value. The p-value for T_on_ is less than 0.05, indicating that the factor is significant, whereas the p-values for T_off_ and WF are greater than 0.05, indicating that there is no noteworthy effect on SR. The F-test shows Table’s F-value at the 5% level of significance is F _(0.05, 2, 2)_ = 19. So, T_on_ is the most important parameter, as shown in Table 7. Even though GV and WF are included, there is no visible impact on SR. The T_off_ is pooled up with error.

**Table 6:** Response table for SR.

Level	Pulse on Time	Pulse off Time	Gap Voltage	Wire Feed
1	**-9.65**	-10.89	-10.76	-10.85
2	-11.07	-10.74	-11.14	-11.07
3	-11.60	**-10.68**	**-10.41**	**-10.40**
Delta	1.95	0.21	0.74	0.67
Rank	1	4	2	3

**Table 7:** ANOVA for SR.

Source of Variation	DF	SS	MS	F	P	C %
Pulse on Time	2	6.11	3.06	86.87	0.01	79.46
Gap Voltage	2	0.81	0.41	11.51	0.08	10.53
Wire Feed	2	0.70	0.35	9.95	0.09	9.10
Pooled Error	2	0.07	0.04			0.91
Total	8	7.69				100.00

### Analysis and discussion of results of kerf

**Fig 9:**
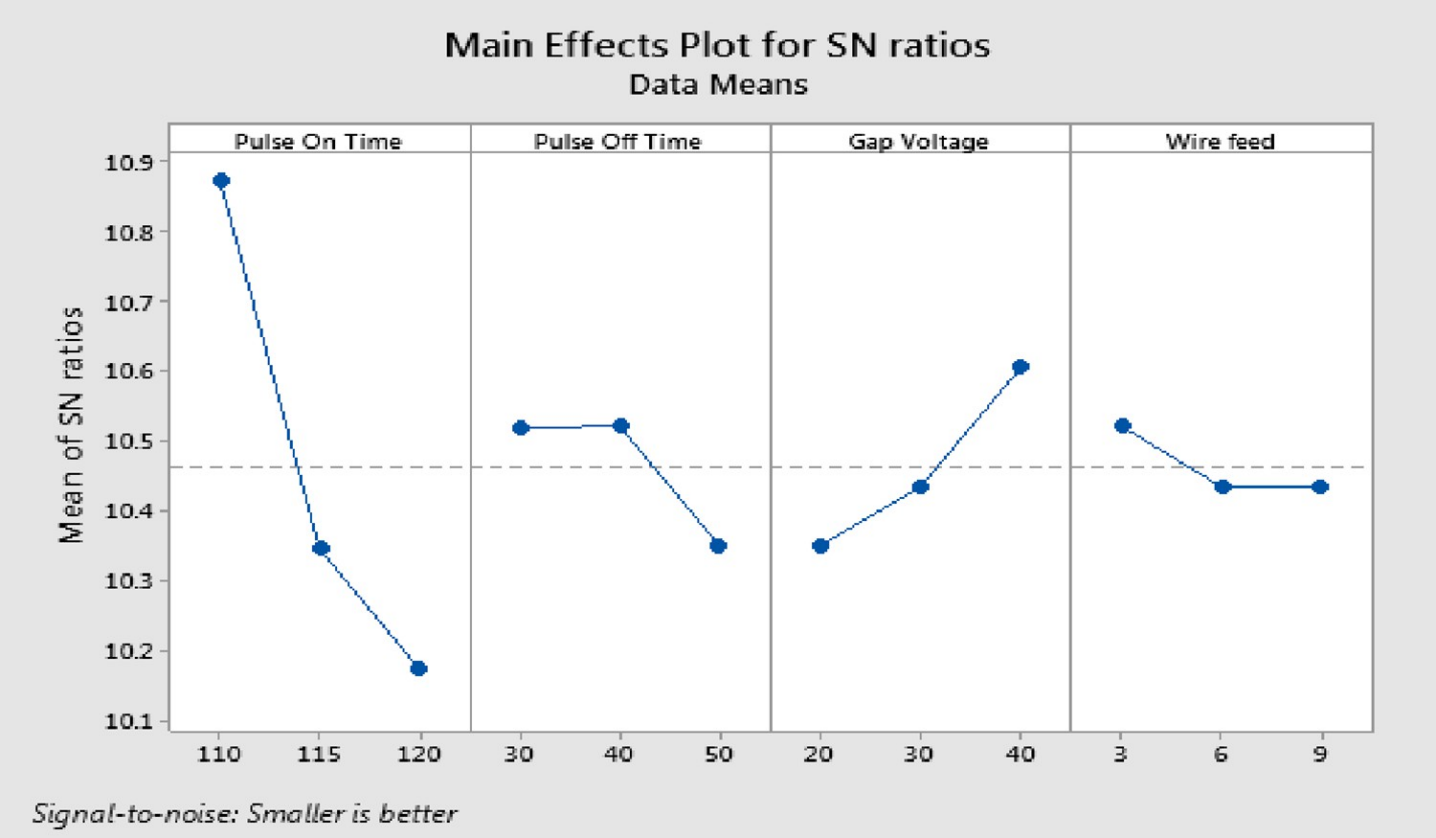
Response graphs for kerf.

Lesser cutting width (Kerf) is found to be more advantageous for better product quality, the "smaller is better" class is selected for S/N calculation. Fig 9 illustrates how the T_on_, T_off_, and WF drop as the K_w_ increases. It gets smaller as the GV gets bigger. The cause is that the discharge energy changes with T_on_, and higher discharge energies produce considerably larger craters, which raise the K_w_ on the work piece. As the GV increases, the average discharge gap widens, resulting in reduced K_w_. Lower K_w_ is also provided by the lower WF [38].

### Selection of optimal levels for kerf

For the purpose of selecting the ideal parameters, the experiment results are assessed. In the Wire EDM phase, Fig 9 and Table 8 demonstrates that the first level of T_on_, second level of T_off_, third level of GV, and first level of WF have the smallest K_w_. R^2^ coefficient is 98.44%, which is an excellent value. The p-value for T_off_, WF, and GV is greater than 0.05, indicating that there was no significant effect on K_w_, whereas the p-value for Pulse on Time is less than 0.05, demonstrating the significance of the factor. The Fisher’s F-test shows at a 95% confidence level, the F-table value is F_(0.05, 2, 8)_ = 19.37. Therefore, the most important parameter, as shown in ANOVA Table 9, is T_on_, even if GV and T_off_ are additionally accounted for in the contribution but have no noticeable impact on K_w_. The WF is a pooled up with the error.

**Table 8:** Response table for kerf.

Level	Pulse on Time	Pulse off Time	Gap Voltage	Wire Feed
1	**10.87**	10.52	10.35	**10.52**
2	10.35	**10.52**	10.43	10.43
3	10.17	10.35	**10.61**	10.43
Delta	0.7	0.17	0.26	0.09
Rank	1	3	2	4

**Table 9:** ANOVA for kerf.

Sources of variation	DOF	SS	MS	F	P	C %
Pulse on Time	2	0.80	0.40	52.6	0.02	81.97
Pulse off Time	2	0.06	0.03	3.83	0.21	5.96
Gap Voltage	2	0.10	0.05	6.74	0.13	10.50
Pooled Error	2	0.02	0.01			1.56
Total	8	0.97				100

### Confirmation experiments

The confirmation experiments, according to Taguchi, is an essential stage in validating the experimental results. Based on the ideal confluence of variables impacting MRR, SR and Kerf, the confirmation experiments were effectively carried out. The tests were run three times to obtain an average value, and the correlation between the actual value and the predicted value was then performed. Table 10 is a summary of the outcomes of the confirmation tests. The best parameters are used to predict the MRR, SR, and K_w_, in the confirmation trials. The experiments’ results are assessed to determine the most important parameters. The best settings for increasing MRR are those at level A_3_, B_1_, C_1_, and D_2_ (T_on_ 120 μs, T_off_ 30 μs, GV 20 V, and WF 6 m/min). While the MRR predicted value is 6.56 mm^3^/min, the MRR experimental result is 6.73mm^3^/min. The predicted and experimental MRR values exhibit remarkable agreement, and the error is 2.53 percent. In order to achieve the lowest SR, the optimum process variables are T_on_ 110 μs, T_off_ 50 μs, GV 40 V, and WF 9 m/min. The experimental SR is 2.76μm, whereas the predicted SR is 2.76 μm. The predicted and experimental SR values exhibit a high degree of agreement. The variables at levels A_1_, B_2_, C_3_, and D_1_ are T_on_ 110 μs, T_off_ 40 μs, GV 40 V, and WF 3 m/min are the optimum machining variables in order to achieve the lowest Kerf. The experimental K_w_ is 0.285 mm and the predicted K_w_ is 0.270 mm. The predicted and experimental K_w_ values are in excellent agreement, and the error is only 2.81%.

**Table 10:** Results of confirmation experiments.

Response	Optimum levels	Experimental value			Average Experimental Value	Predicted Value	Error %
		Trial 1	Trial 2	Trial 3			
MRR (mm^3^/min)	A_3_B_1_C_1_D_2_	6.63	6.87	6.69	6.73	6.56	2.53
Surface Roughness (μm)	A_1_B_3_C_3_D_3_	2.76	2.77	2.75	2.76	2.76	0
Kerf Width (mm)	A_1_B_2_C_3_D_1_	0.278	0.290	0.287	0.285	0.277	2.81

### SEM analysis of WEDMed surfaces

In order to assess the surface integrity, the SEM study on the topography of the WEDM machined LM5 aluminium alloy surfaces was conducted using the optimum surface roughness process variables: T_on_ 110 μs, T_off_ 50 μs, GV 40 V, and WF 9 m/min.

**Fig 10:**
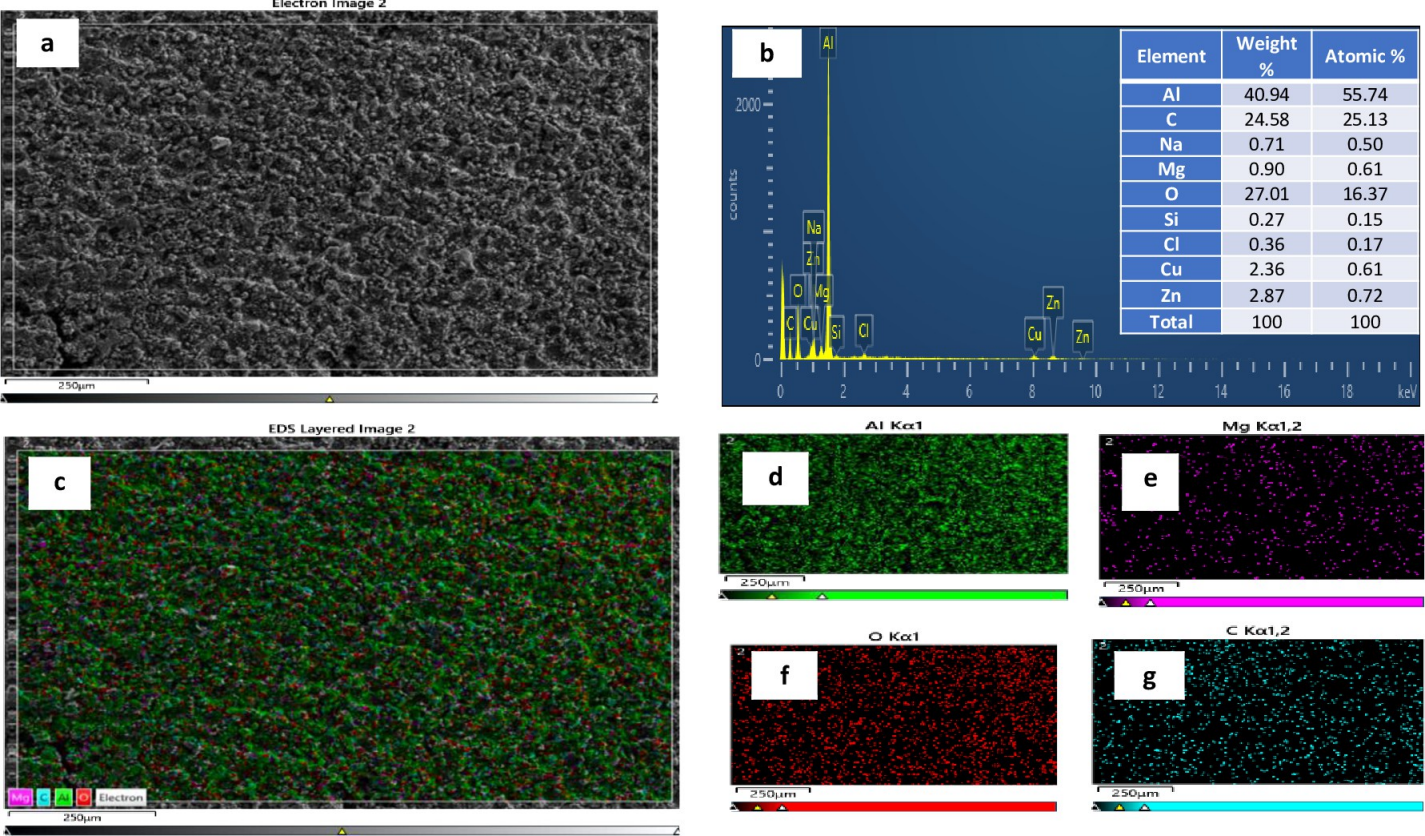
WEDM–Machined Surface a) SEM image b) EDAX c) EDX mapping d) Al e)Mg f) O g) C.

The SEM micrograph of WEDM machined surface at machining parameters A_1_B_3_C_3_D_3_ in the orthogonal array of experiment is shown in Fig 10A, it is clear that the size of the crater depend on the discharge heat energy or in other words, on the gap voltage and pulse on time values. Higher Gap voltage causes an increase in discharge heat energy at the point where the discharge takes place. At this point, a pool of molten metal is formed and is overheated. The overheated molten metal evaporates forming gas bubbles that explode when the discharge ceases, taking molten material away. The result is the formation of crater. Successive discharges that have a random nature will result in the formation of globules of debris, shallow craters, pockmarks and cracks [39, 40].

The surface morphology of WEDMed LM5 alloy was studied by field emission scanning electron microscopy (FESEM). To determine the elemental composition, an EDX analysis was performed. Fig 10B shows the EDX image of machined LM5 Al alloy and the atomic weight. The formation of carbon is represented by distinct peaks following aluminium. The XRD analysis revealed the presence of all the alloying elements, which was further confirmed by the EDX analysis. The EDAX of machined surface (recast layer) shows oxygen, carbon, copper and zinc. These are formed due to the dielectric fluid and tool material (brass wire). As shown in Fig 10C–10G, EDX mapping shows the quantity of each element inside the reaction product.

The fundamental concept of the WEDM technique is the generation of electric sparks between the work piece and the wire electrode. These electrical discharges release a large quantity of heat at temperatures ranging from 8000 to 12,000°C, resulting in melting and evaporation of work piece material at the nearby surface layers. The heat also melts the dielectric medium (de-ionized water) and creates high-pressure waves that wash away the melted and/or evaporated metal from the work piece. Throughout the WEDM process, dielectric fluid is continuously supplied to transport the eroded metal apart. As a result of water’s strong thermal conductivity, the top surface cools and un-expelled material re-solidifies at a rapid pace. This re-solidified layer, known as a recast layer, is often highly fine-grained, hard, brittle, and structurally distinct from its parent material. The creation of these layers is determined by the process parameters as well as the work piece’s chemical composition and heat conductivity.

**Fig 11:**
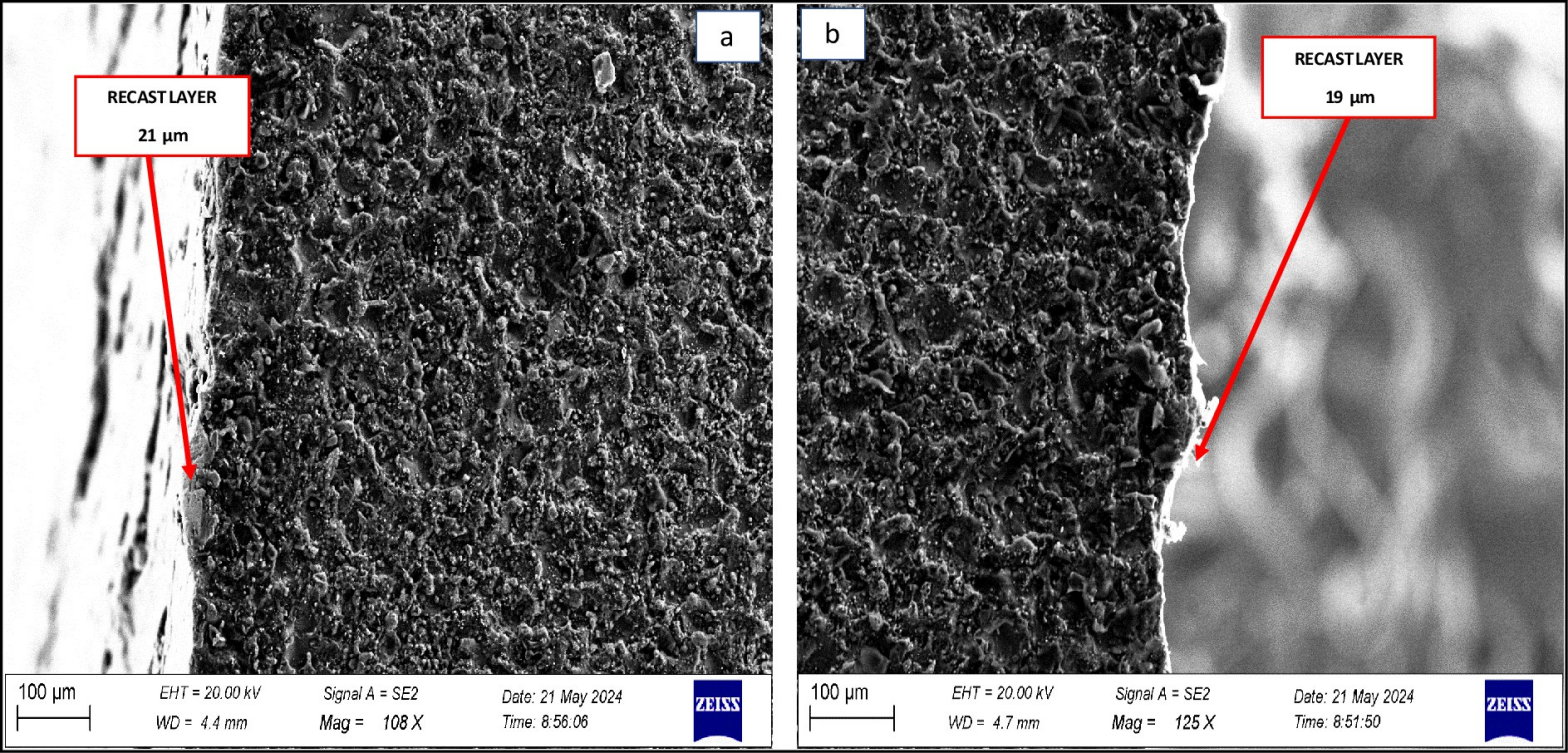
Recast layer.

The recast layer as shown in Fig 11 is generated at a slower cooling rate from the outermost layer, allowing the melted material to re-solidify fast and without grain boundaries. The heat-affected zone appears somewhat distinct in colour since it does not melt but is heated throughout the machining process. Zhang et al. [41] investigated different types of di-electrics and determined the recast layer created by water-in-oil emulsion dielectric, which has larger surface roughness and thickness than the kerosene and de-ionized water dielectrics. The results have revealed that the thickness of the recast layer increases with increasing peak current and decreases by using de-ionized water as the dielectric.

### XRD analysis of WEDMed surface

**Fig 12:**
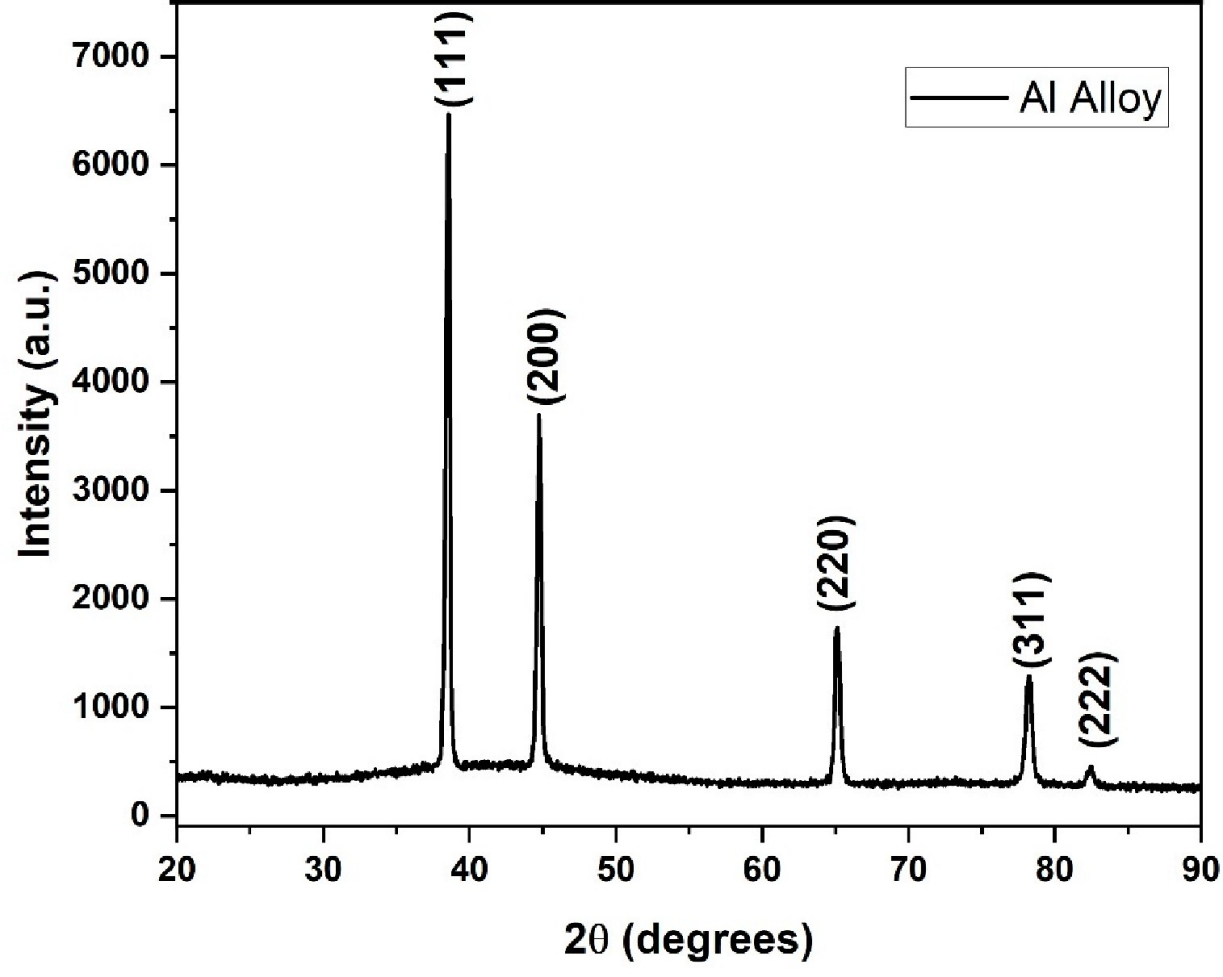
XRD of WEDMed aluminium alloy LM5.

It is observed that the peaks corresponding to the sample LM5 alloy have good agreement with the JCPDS card 04–0787. So, it is concluded that the crystal structure and elemental composition of the LM5 alloy have not disturbed even after the WEDM machining process. The average crystallite size is 27 nm and the phase is Fm-3m [2 2 5]. Further, it is observed that the lattice parameters are equal which reflects the cubic structure and the primitives are a = b = c = 4.054Ǻ. The XRD image of Wire Electro Discharge machined specimen is shown in Fig 12.

### Effect of process parameters on responses (MRR, SR, and K_w_)

#### Effect of T_on_ on responses

**Fig 13:**
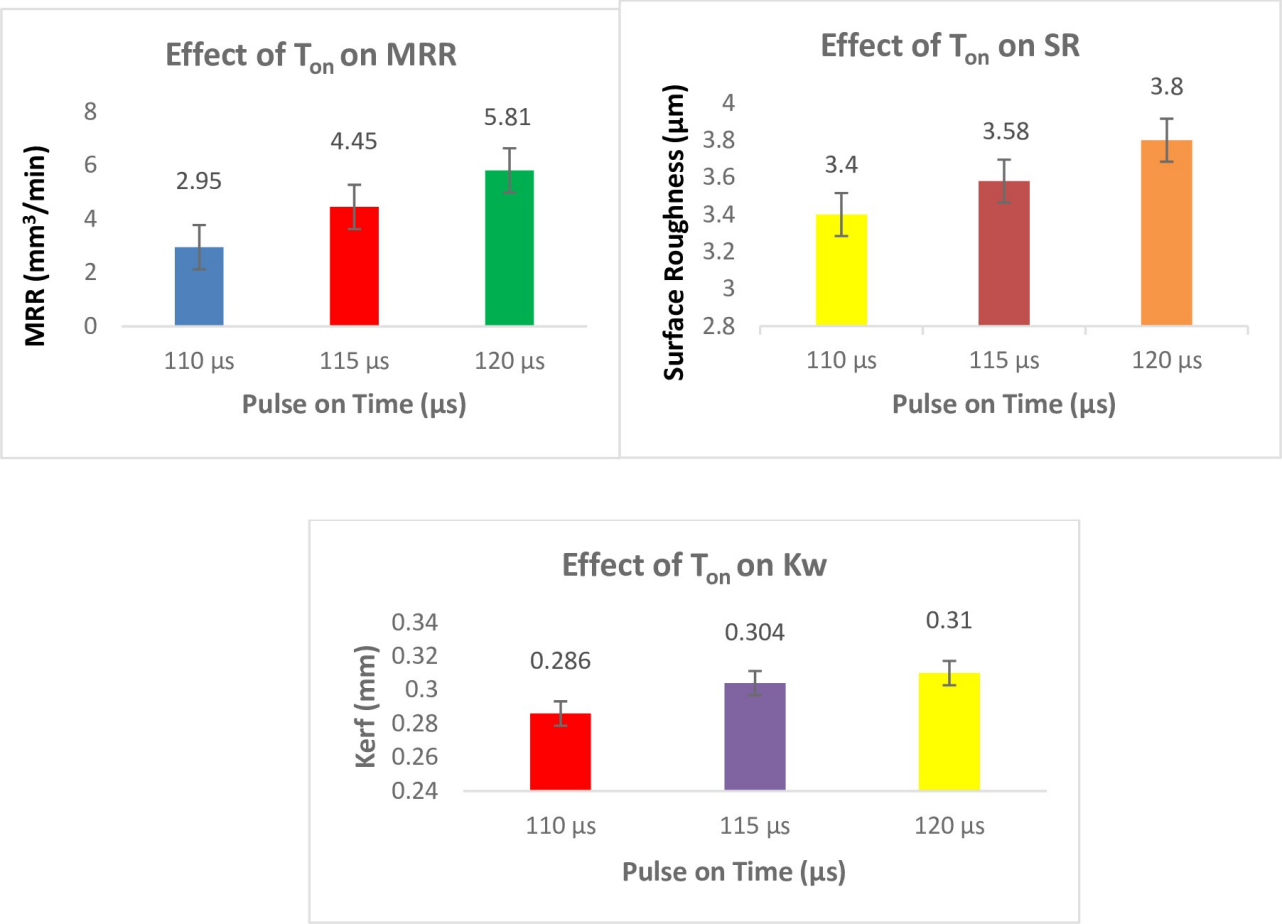
Effect of Pulse on Time on responses.

Fig 13 shows the effect of T_on_ on responses. Longer T_on_ emits higher discharge energy, which causes stronger explosions, deeper craters on the surface of the work piece, and higher MRR. Deep craters suggest high rates of MRR and subpar surface quality. Larger values of T_on_ should be used to get better MRR. The T_on_ that produces the highest MRR is 120 μs [42]. As gap voltage (GV) decreases and pulse-on time (T_on_) increases, surface roughness increases. The size and shape of surface craters, which are influenced by discharge energy and the re-deposition of melted material on the work surface, are the main factors that define SR in WEDM. Surface roughness increases when the servo voltage is decreased and the pulse-on-time is increased because this increases the discharge energy across the electrodes and creates a deep erosion crater on the work piece’s surface. There is a significant chance that molten material will re-deposit on the work surface at high discharge energies [43].

According to the findings, raising the T_on_ causes a greater thermal energy transfer from the wire to the work piece, which increases cutting velocity. As the T_on_ falls, SR lowers. SR has an impact on the wire electrical discharge machining finish cut. Experiments have shown that lowering the T_on_ and the discharge current together can reduce SR. A large number of pertinent investigations found that as discharge energy increased the Wire EDMed surface roughness because of more craters were created, which led to higher SR values on the work piece. Increasing the pulse on time, the single pulse discharge electric energy increases thickness of cutting surface discharge, however the electrical erosion products’ duration of discharge reduces proportionately, leading to burns on the cutting surface and the generation of adhesive substance there. These factors collectively influence the increase in surface roughness [44]. Kerf reduces when T_on_ declines. K_w_ can be used to gauge the material’s dimensional correctness. Experiments show that lowering the discharge current and pulse duration can lower Kerf. Furthermore, since K_w_ is based on the size of the spark crater, related research discovered that T_on_ is the main variable affecting K_w_. The discharge energy must be kept at a low level by employing short pulse duration in order to produce flat craters [45].

#### Effect of T_off_ on responses

**Fig 14:**
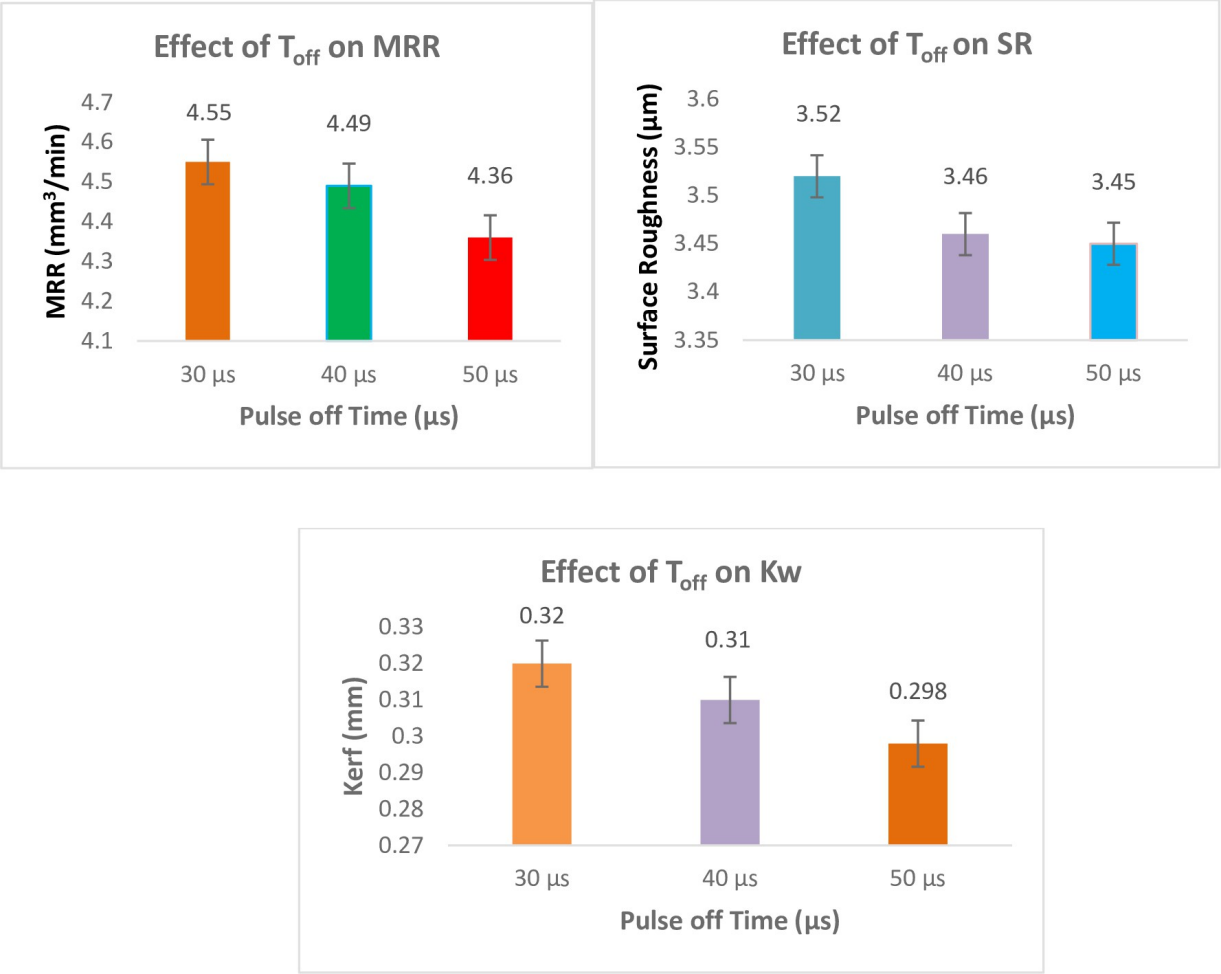
Effect of Pulse off Time on responses.

Fig 14 shows the effect of T_off_ on responses. Results demonstrate that MRR declines as T_off_ rises. Due to longer non-cutting times, greater T_off_ causes a drop in MRR. A wider gap is produced by a longer T_off_, but it also offers a longer flushing time to remove the debris from the gap. Usually an extended T_off_ was used to stop wire rupturing or to stop the abnormal process. It may be concluded that higher T_off_ causes lower SR since the non-cutting time increases [46]. Increasing the T_off_ value extends the duration between 2 successive sparks, resulting complete flushing of carbide debris out of the spark gap, low re-deposition of degraded material, and low SR. Surface roughness has a slight tendency to decrease with increased WF. Increasing wire feed allows carbide debris to easily escape from the spark gap, resulting in re-cast layer. To get an excellent surface finish, keep the electrical discharge energy to a minimum by choosing minimal T_on_ and an excessive T_off_ [47]. The quantity of single pulse discharge energy is unaffected by an increase in pulse interval; rather, it only affects the length of discharge time per unit of time. Consequently, the discharge duration per unit time doubles and the cutting speed decreases linearly as the pulse interval grows. It has been found that raising the T_off_ causes the MRR to decrease. This action enhances the procedure by enabling a more effective flush of debris into the gap. 50 μs is the ideal pulse off time for lowering SR and K_w_ [48].

#### Effect of GV on responses

**Fig 15:**
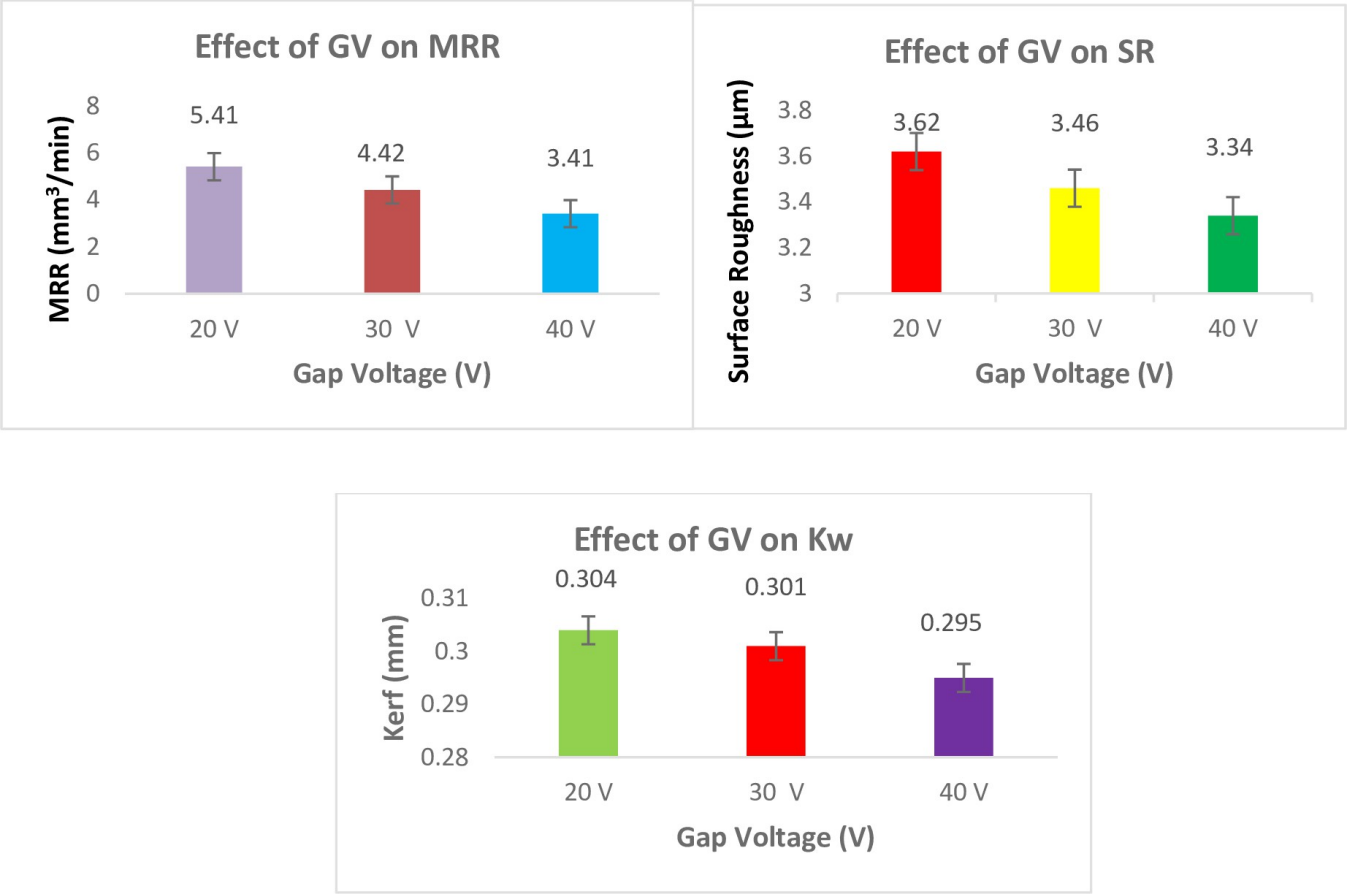
Effect of Gap Voltage on responses.

Fig 15 shows the effect of GV on responses. The findings indicate that the MRR rises as the GV falls. 20 V is the ideal gap voltage for increasing MRR. It’s important to note that 20 V is the base alloy’s ideal voltage. Due to a greater electric field, spark discharge actually occurs beneath the same gap more frequently when voltage increases. Less voltage can provide enough energy to melt the dielectric particles in the vicinity. The SR reduces as the GV rises. This occurrence is remarkable. Lower GV may have sufficient energy to melt the re-solidified particles in the vicinity, which remain on the machined surface and produce a large number of projecting peaks. On the other side, a smoother surface is produced by high voltage [49]. Melting of the component’s surface is determined by the thermal conductivity of the work piece material and the quantity of energy used per spark that is assumed to be proportional to T_on_ and GV. Enhancing the pulse on time (T_on_) generates more heat at the work surface, improving the cutting speed.

Lowering Gap voltage decreases the spark gap, resulting in fast and significant ionization of the dielectric fluid, causing greater melting of the work material and hence increases cutting speed [50]. Increasing electrostatic force brought on the increasing GV causes wire wrapping during the discharge process. The SR lowers as the gap voltage rises. 40 V is the ideal GV for getting lower K_w_. Sparks that form at the conducting phase and produce melting or possible evaporation also contribute to the craters on the machined surface. It goes without saying that huge K_w_ are caused by high crater diameters [51, 52].

#### Effect of WF on responses

**Fig 16:**
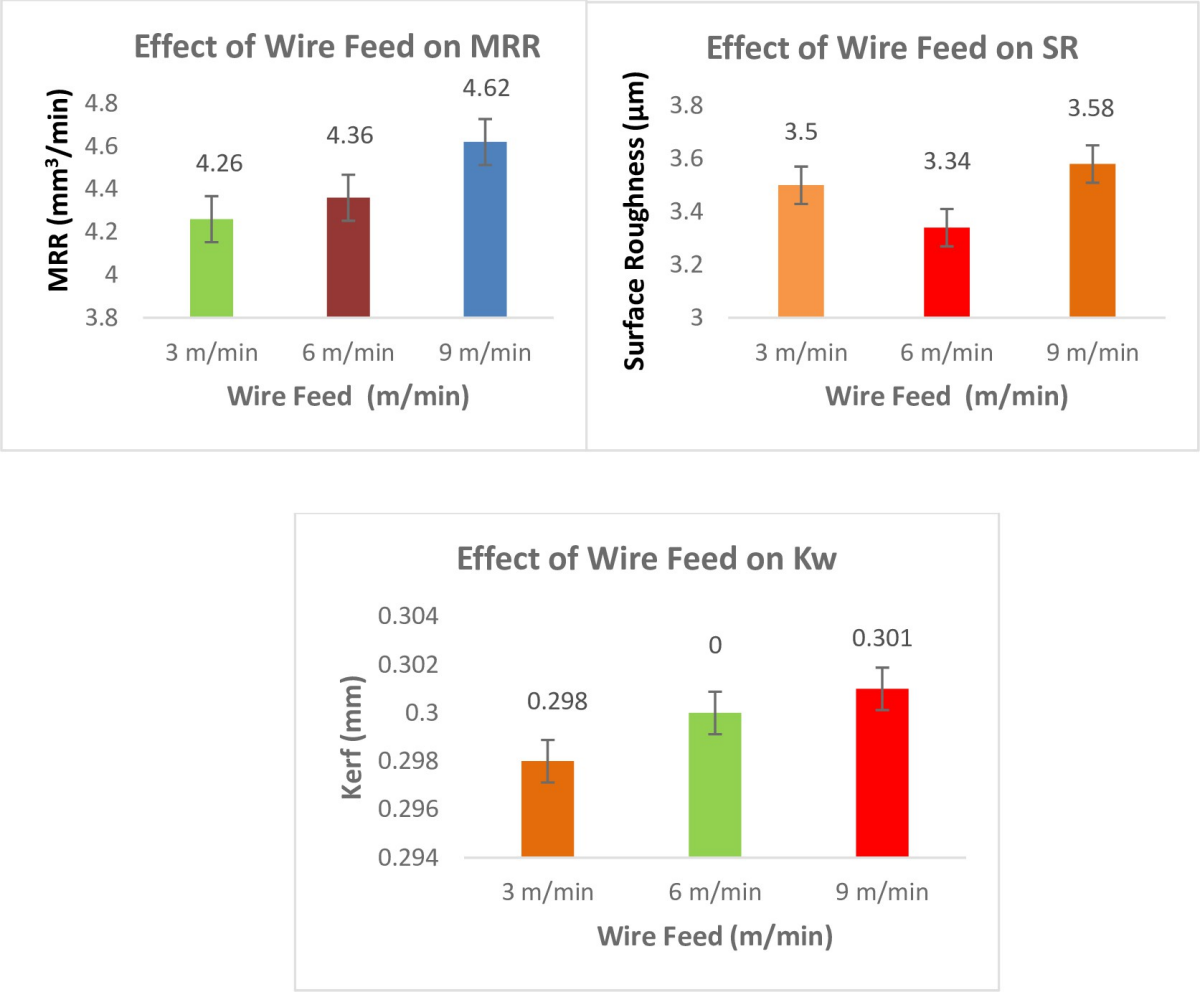
Effect of Gap Voltage on responses.

Fig 16 shows the effect of WF on responses. The WF ought to be selected in a way that prevents wire breaking. As the wire feed accelerates, the MRR ascend. For maximizing MRR, a wire feed setting of 6 m/min is ideal. In a word, this investigation’s findings supported those in the literature [53]. At high wire feed values, the increase in cutting speed is particularly noticeable. Higher discharge energy causes increased melting and evaporation of the work material, resulting in the release of a significant quantity of carbide debris, which coagulates in the spark gap and so influences the machining process through the generation of arcs. Raising the wire feed rate allows the eroded material to escape the spark gap more easily and quickly [54]. Even though the research was done on a variety of materials, the outcomes were reliable. It is important to choose a WF that wire won’t break. As the WF grows, so does the cutting speed. For a minimum SR, a wire feed setting of 6 m/min is ideal. Briefly stated, the findings of this investigation were consistent with those discovered in the literatures. The investigation was conducted on a variety of materials, but the outcomes were constant. A non-critical metric is wire feed. The ideal WF for the lowest K_w_ is 3 m/min. Increasing the WF rate might make the wire less rigid during discharge, which would reduce the amount of wire that would wind back during sparks and reduce the destructive power of the sparks on the work piece surface, leading to a higher K_w_ [55, 56].

### Mathematical models of LM5 aluminium alloy

Multiple linear regression (MLR) models frequently provide a suitable illustration of a more complex structure within particular ranges of the independent variables. The generalization model of MLR analysis describes the relationship among the response and the independent variables, and the estimated response is calculated using the generalized simple regression equation. The constants were calculated using the linear regression analysis method using MINITAB software.

Mathematical models for MRR, SR and K_w_ for LM5 alloy are developed using Linear Regression are shown in Eqs 1–3.


$$MRR=–26.38+0.2857\;T_{on}+0.01473\;T_{off}–0.10002\;GV+0.0585\;WF$$ (1)

$$SR=–4.81+0.0760\;T_{on}–0.00292\;T_{off}–0.00575\;GV–0.0278\;WF$$ (2)

$$Kerf=0.0225+0.002400\;T_{on}+0.000300\;T_{off}–0.000450\;GV+0.000500\;WF$$ (3)

From the ANOVA of the Regression, the R^2^ value of MRR is 99.7, the R^2^ value of SR is 82.6 and the R^2^ value of Kerf is 91.4.

The aforementioned mathematical model for MRR, SR and kerf is critical for selecting machining settings while machining LM5 Aluminium alloy using WEDM. According to Eqs 1 and 3, MRR and Kerf are directly correlated to T_on_, T_off_ and WF, but inversely correlated to gap voltage. Eq 2 demonstrates that SR is directly proportional to T_on_ and inversely proportional to T_off_, GV and WF.

From Eq (1), it is seen that T_on_, T_off_ and WF are directly proportional and GV is inversely proportional to MRR. The percentage deviation of MRR values between the experimental and predicted values are reported in Table 11. Referring to Table 11 on MRR model, it can be seen lesser deviation occurs between experimental and predicted values. The average absolute error for MRR is 1.69%. These deviations could be attributed to limitation of the modeling not accounting for interactive influences [57, 58].

**Table 11:** Experimental and predicted values of MRR, SR and kerf.

Ex No	A	B	C	D	Experimental	Predicted	%	Experimental	Predicted	%	Experimental	Predicted	%
	T_on_ (μs)	T_off_ (μs)	GV (V)	WF (m/min)	MRR (mm3/ min)	MRR	Deviation	SR	SR	Deviation	kerf	kerf	Deviation
1	110	30	20	3	3.61	3.66	-1.5	3.1	3.26	-5.29	0.286	0.288	-0.7
2	110	40	30	6	2.98	2.99	-0.22	3.27	3.09	5.39	0.286	0.288	-0.7
3	110	50	40	9	2.26	2.31	-2.18	2.76	2.92	-5.93	0.286	0.288	-0.7
4	115	30	30	9	4.5	4.44	1.26	3.63	3.42	5.79	0.304	0.299	1.81
5	115	40	40	3	3.4	3.24	4.72	3.45	3.5	-1.44	0.295	0.294	0.34
6	115	50	20	6	5.52	5.56	-0.77	3.66	3.5	4.31	0.313	0.308	1.76
7	120	30	40	6	4.56	4.7	-2.98	3.83	3.83	0.11	0.304	0.305	-0.16
8	120	40	20	9	7.09	7.02	1	3.63	3.83	-5.45	0.313	0.318	-1.6
9	120	50	30	3	5.78	5.82	-0.61	3.96	3.91	1.31	0.313	0.314	-0.16

From Eq (2), it is seen that GV, T_off_ and WF are directly proportional and T_on_ is inversely proportional to SR. The percentage deviations of SR values between the experimental and predicted values are reported in Table 11. Referring to Table 11 on SR model, it can be seen lower order deviation between experimental and predicted values occurs. The average absolute error for SR is 3.89%. These deviations could be attributed to limitation of the modeling not accounting for interactive influences.

From Eq (3), it is seen that T_on_, T_off_ and WF are directly proportional and GV is inversely proportional to kerf. The percentage deviation of kerf values between the experimental and predicted values are reported in Table 11. Referring to Table 11 on kerf model, it can be seen minimum deviation between experimental and predicted values occurs. The average absolute error for kerf is 0.88%.

## Conclusions

### The LM5 aluminium alloy was successfully produced via the stir casting technique. The subsequent outcomes were attained

- The microscopic and SEM images show the microstructure of the LM5 Al alloy and EDX shows the constituents.
- The Pulse on Time (68.25%) and Gap Voltage (31.68%) has the greatest statistical effects on MRR. The error associated with the ANOVA is 0.07%, indicating a 95% confidence level.
- The most significant statistical impact on SR is possessed by the Pulse on Time (79.46%). The ANOVA error for SR is 0.91%, indicating a 95% confidence level.
- The greatest statistical influence on K_w_ is by the Pulse on Time (81.97%). The 95% confidence level is shown by the error associated with the ANOVA for K_w_, is 1.56%.
- The error % associated with the predicted and experimented values of the MRR is 2.53%, SR is 0 and K_w_ is 2.81%, based on the confirmation experiments.
- The EDS of the machined surface (recast layer) shows oxygen, carbon, copper and zinc. These are formed due to the dielectric fluid and tool material (brass wire).
- The average absolute error for MRR is 1.69%, for SR is 3.89% and for kerf is 0.88%, based on mathematical (linear regression) models.
- The predicted and experimental values based on the mathematical models are very close to each other confirms that the Taguchi’s Signal to Noise ratio analysis is the most appropriate for single objective optimization of responses.

The findings of this investigation promises to enhance precision and surface quality in wire electro-discharge machining of LM5 aluminium alloy in today’s production processes.

## References

[1] Wang L., Makhlouf M. and Apelian D., 1995. Aluminium die casting alloys: alloy *composition*, *microstructure*, *and properties-performance relationships*. *International* materials reviews, 40(6): 221–238.

[2] Rajan R., Kah P., Mvola B. and Martikainen J., 2016. Trends in aluminium alloy development and their joining methods. *Reviews on Advanced Materials Science*, 44(4)

[3] Liptakova T., Zatkalíková V., Alaskari A.M. and Malcho M., 2019. Dominant factors affecting erosion-corrosion resistance of aluminium–brass pipelines. Journal of Engineering Research, 7(4).

[4] Boggarapu V., Sreekanth P.R. and Peddakondigalla V.B., 2023. Microstructure, Mechanical and Tribological Properties of Al/Cu Functionally Graded Material Fabricated through Powder Metallurgy. Journal of Engineering Research, p.100119.

[5] Ananth S., Prakash J.U., Juliyana S.J., Rubi C.S. and Sadhana A.D., 2021. Effect of process parameters on WEDM of Al–Fly ash composites using Taguchi Technique. *Materials Today*: *Proceedings*, 39, pp.1786–1790.

[6] Ho K.H., Newman S.T., Rahimifard S. and Allen R.D., 2004. State of the art in wire electrical discharge machining (WEDM). *International Journal of Machine Tools and Manufacture*, 44(12–13), pp.1247–1259.

[7] Saravanan R., Anbuchezhiyan G., Mamidi V.K. and Kumaran P., 2022. Optimizing WEDM parameters on nano-SiC-Gr reinforced aluminium composites using RSM. *Advances in Materials Science and Engineering*, 2022. doi: 10.1155/2022/1612539

[8] Natarajan K., Ramakrishnan H., Gacem A., Vijayan V., Karthiga K., Ali H.E., et al. 2022. Study on optimization of WEDM process parameters on stainless steel. *Journal of Nanomaterials*, 2022.

[9] Rouniyar A.K. and Shandilya P., 2021. Semi-empirical modeling and optimization of process parameters on overcut during MFAPM-EDM of Al6061 alloy. Proceedings of the Institution of Mechanical Engineers, Part E: Journal of Process Mechanical Engineering, 235(6), pp.1784–1796. doi: 10.1177/09544089211015890

[10] Kar S. and Sahoo R., 2023. Viability and performance assessment of bipolar linear self-servo mechanism-based Maglev electrical discharge machining by processing Al-6062 alloy. Proceedings of the Institution of Mechanical Engineers, Part E: Journal of Process Mechanical Engineering, p.09544089231207685.

[11] Mahapatra S.S. and Patnaik A., 2006. Parametric optimization of wire electrical discharge machining (WEDM) process using Taguchi method. Journal of the Brazilian Society of Mechanical Sciences and Engineering, 28, pp.422–429.

[12] Verma R.K., Patel D. and Chopkar M.K., 2023. Wear behavior investigation of Al-B_4_C functionally graded composite through Taguchi’s design of experiment. Journal of Engineering Research, p.100095.

[13] Sarkar S., Sekh M., Mitra S. and Bhattacharyya B., 2011.A novel method of determination of wire lag for enhanced profile accuracy in WEDM. *Precision Engineering*, 35(2), pp.339–347. doi: 10.1016/j.precisioneng.2011.01.001

[14] Arunnath A., Madhu S. and Tufa M., 2022. Experimental investigation and optimization of material removal rate and tool wear in the machining of aluminium-boron carbide (Al-B4C) nanocomposite using EDM process. *Advances in Materials Science and Engineering*, 2022.

[15] Salavaravu L. and Dumpala L., 2022. Effects of process parameters on mechanical and metallurgical properties of AA5083 weld bead and optimization by using Taguchi based Grey Relational Analysis and ANOVA of submerged friction stir welding. Journal of Engineering Research (2307–1877), 10.

[16] Zafar M.M. and Toor Z.S., 2023. Experimental and numerical investigation of electrochemical and tribological behavior of aluminium alloys. Journal of Engineering Research, p.100134.

[17] Kumar N., Kumari S., Abhishek K., Nandi G. and Ghosh N., 2022. Study on various parameters of WEDM using different optimization techniques: A review. *Materials Today*: *Proceedings*, 62, pp.4018–4024.

[18] Kansal H.K., Singh S. and Kumar P., 2007. Technology and research developments in powder mixed electric discharge machining (PMEDM). *Journal of materials processing technology*, 184 *(*1–3*)*, *pp*.32–41.

[19] Debnath T. and Patowari P.K., 2019. Fabrication of an array of micro-fins using Wire-EDM and its parametric analysis. Materials and Manufacturing Processes, 34(5), pp.580–589.

[20] Sarmah P. and Patowari P.K., 2023. Machinability study of Al 6063-based MMCs with SiC reinforcement particles using WEDM process. Materials and Manufacturing Processes, 38(7), pp.783–796.

[21] Sahoo R., Kar S. and Patowari P.K., 2020. Experimental study on Titanium diamond by fabricating micro-holes using Micro-EDM with Taguchi’s method and response surface method. Engineering Research Express, 2(4), p.045026.

[22] Sahoo R., Singh N.K. and Bajpai V., 2023. A novel approach for modeling MRR in EDM process using utilized discharge energy. Mechanical Systems and Signal Processing, 185, p.109811.

[23] Sahoo R., Singh N.K. and Bajpai V., 2023. Approach towards green manufacturing in Maglev EDM using different biodegradable dielectrics at variable discharge conditions. Journal of Cleaner Production, 430, p.139623.

[24] Ye L., Qian J., Haitjema H. and Reynaerts D., 2022. On-machine chromatic confocal measurement for micro-EDM drilling and milling. Precision Engineering, 76, pp.110–123.

[25] Juliyana S.J., Prakash J.U., Salunkhe S., Hussein H.M.A. and Gawade S.R., 2022. Mechanical Characterization and Microstructural Analysis of Hybrid Composites (LM5/ZrO_2_/Gr). *Crystals*, 12(9), p.1207.

[26] Jebarose Juliyana S. and Prakash J.U., 2022. Optimization of machining parameters for wire EDM of AMCs (LM5/ZrO_2_) using Taguchi technique. *INCAS Bulletin*, 14(1), pp.57–68.

[27] Jebarose Juliyana S., and Prakash J.U., 2020. Drilling parameter optimization of metal matrix composites (LM5/ZrO2) using Taguchi Technique. *Materials Today*: *Proceedings*, 33, pp.3046–3050.

[28] Jebarose Juliyana S., Udaya Prakash J., Čep R. and Karthik K., 2023. Multi-Objective Optimization of Machining Parameters for Drilling LM5/ZrO2 Composites Using Grey Relational Analysis. *Materials*, 16(10), p.3615. doi: 10.3390/ma16103615 37241242 PMC10221591

[29] Jebarose Juliyana S., Prakash J.U., Sadhana A.D. and Rubi C.S., 2022. Multi-objective optimization of process parameters of wire EDM for machining of AMCs (LM5/ZrO2) using grey relational analysis. *Materials Today*: *Proceedings*, 52, pp.1494–1498.

[30] Juliyana S.J., Prakash J.U. and Salunkhe S., 2022.Optimisation of wire EDM process parameters using Taguchi technique for machining of hybrid composites. *International Journal of Materials Engineering Innovation*, 13(3), pp.257–271.

[31] Udaya Prakash J; Jebarose Juliyana S.; Salunkhe S.; Gawade S.R.; Nasr E.S.A.; Kamrani A.K., 2023. Mechanical Characterization and Microstructural Analysis of Stir-Cast Aluminum Matrix Composites (LM5/ZrO2). *Crystals*, 13, 1220.

[32] Wang B., Liu X., Wang J., Li Q., Liu K., & Zhang M., 2022. Uncovering the effects of Ce and superheat temperature on Fe-rich intermetallic and microporosity formation in aluminum alloy. *Materials Characterization*, 193, 112226.

[33] Balan A.S.S. and Giridharan A., 2017. A progress review in wire electrical discharge machining process. International Journal of Automotive and Mechanical Engineering, 14, pp.4097–4124.

[34] Kousik Kumaar R., Somasundara Vinoth K. and Kavitha M., 2022. Dry sliding wear performance of AA7075/MoS_2_ composite materials. Journal of Engineering Research (2307–1877), 10

[35] Paulson D.M., Saif M. and Zishan M., 2023. Optimization of wire-EDM process of titanium alloy—Grade 5 using Taguchi’s method and grey relational analysis. *Materials Today*: *Proceedings*, 72, pp.144–153.

[36] George J., Philip J.T., Mathew J. and Manu R., 2022. Prediction and analysis of material removal rate and white layer thickness during wire electrical discharge turning (WEDT) process. *CIRP Journal of Manufacturing Science and Technology*, 39, pp.210–222

[37] Ram H.S., Uthayakumar M., Kumar S.S., Kumaran S.T. and Korniejenko K., 2022.Modelling Approach for the Prediction of Machinability in Al6061 Composites by Electrical Discharge Machining. *Applied Sciences*, 12(5), p.2673.

[38] Sahu S.K., Anand M.V., Kumar T.C.A., Kumar A., Prasad G.S. and Niraj V.V., 2023. Kerf width analysis of wire electrical discharge machining of titanium alloy. *Materials Today*: *Proceedings*.

[39] Kumar A., Kumar V. and Kumar J., 2016. Surface crack density and recast layer thickness analysis in WEDM process through response surface methodology. *Machining Science and Technology*, 20(2), pp.201–230.

[40] Azam M., Jahanzaib M., Abbasi J.A., Abbas M., Wasim A. and Hussain S., 2016. Parametric analysis of recast layer formation in wire-cut EDM of HSLA steel. *The International Journal of Advanced Manufacturing Technology*, 87, pp.713–722.

[41] Zhang Y., Liu Y., Ji R. and Cai B., 2011. Study of the recast layer of a surface machined by sinking electrical discharge machining using water-in-oil emulsion as dielectric. *Applied surface science*, 257(14), pp.5989–5997.

[42] Chaudhari R., Shah H., Ayesta I., de Lacalle L.L. and Vora J., 2022. Experimental investigations and optimization of WEDM parameters using Taguchi analysis of pure titanium. In *Recent Advances in Mechanical Infrastructure*: *Proceedings of ICRAM 2021* (pp. 349–358). Springer Singapore.

[43] Sharmila B. and Selvakumar G., 2022. Investigations on the effect of dielectric medium and WEDM parameters on surface characteristics of Al 7068 (ordnance aluminium) alloy. Surface Topography: Metrology and Properties, 10(3), p.035031.

[44] Han F., Jiang J. and Yu D., 2007. Influence of machining parameters on surface roughness in finish cut of WEDM. *The International Journal of Advanced Manufacturing Technology*, 34, pp.538–546.

[45] Li W., Zhao L., Wang G. and Zhou C., 2023. Manufacturing of Mold Cavity of Micro-double Gear by WEDM. In Journal of Physics: Conference Series (Vol. 2459, No. 1, p. 012065). IOP Publishing.

[46] Bisaria H. and Shandilya P., 2023. Surface integrity of NiTi-SMA during WEDM: effect of spark parameters. *Materials and Manufacturing Processes*, pp.1–9.

[47] Sen R., Choudhuri B., Barma J.D. and Chakraborti P., 2020. Surface integrity study of WEDM with various wire electrodes: Experiments and analysis. Machining Science and Technology, 24(4), pp.569–591.

[48] Sharma N., Gupta R.D., Khanna R., Sharma R.C. and Sharma Y.K., 2023. Machining of Ti-6Al-4V biomedical alloy by WEDM: Investigation and optimization of MRR and Rz using grey-harmony search. *World Journal of Engineering*, 20(2), pp.221–234.

[49] Chaubey S.K. and Gupta K., 2023. A review on Wire-EDM of bio titanium. *Reports in Mechanical Engineering*, 4(1), pp.141–152.

[50] Sarala Rubi C., Udaya Prakash J., Jebarose Juliyana S., Robert Čep, Sachin Salunkhe, Kouril K., et al. 2024. Comprehensive review on wire electrical discharge machining: a nontraditional material removal process. *Frontiers in Mechanical Engineering*,10:1322605.

[51] Raj A., Misra J.P., Khanduja D., Saxena K.K. and Malik V., 2022. Design, modeling and parametric optimization of WEDM of Inconel 690 using RSM-GRA approach. *International Journal on Interactive Design and Manufacturing (IJIDeM)*, pp.1–11.

[52] Babu R.R., Subhair S. and Siva S., 2018. Optimization of process parameter in WEDM for Stainless Steel 316 by Using Taguchi Method. International Journal of Engineering Research & Technology (IJERT).

[53] Prakash J.U., Sivaprakasam P., Garip I., Juliyana S.J., Elias G., Kalusuraman G., et al. 2021. Wire electrical discharge machining (WEDM) of hybrid composites (Al-Si12/B4C/fly ash). *Journal of Nanomaterials*, 2021, pp.1–10.

[54] Kumar A., Kumar D.V. and Kumar D.J., 2011. A review on the state of the art in wire electric discharge machining (WEDM) process. International Journal of Mechanical Engineering Research and Development (IJMERD), 1(1).

[55] Patra D.R., Rout I.S. and Sahoo M., 2015. Optimization of WEDM parameters using Taguchi method for higher material removal rate on EN31 steel. International Journal of Engineering Research and Applications, 5(6), pp.57–62.

[56] Ehsan Asgar M. and Singh Singholi A.K., 2018. September. Parameter study and optimization of WEDM process: A Review. In *Iop conference series*: *Materials science and engineering* (Vol. 404, p. 012007). IOP Publishing.

[57] Muthukumar V., Rajesh N., Venkatasamy R., Sureshbabu A. and Senthilkumar N., 2014. Mathematical modeling for radial overcut on electrical discharge machining of Incoloy 800 by response surface methodology. *Procedia Materials Science*, 6, pp.1674–1682.

[58] Muthukumar V., Suresh Babu A., Venkatasamy R. and Senthil Kumar N., 2015. An accelerated particle swarm optimization algorithm on parametric optimization of WEDM of die-steel. *Journal of The Institution of Engineers (India)*: *Series C*, 96, pp.49–56.

